# Timing of Repetitive Transcranial Magnetic Stimulation Onset for Upper Limb Function After Stroke: A Systematic Review and Meta-Analysis

**DOI:** 10.3389/fneur.2019.01269

**Published:** 2019-12-03

**Authors:** Eline C. C. van Lieshout, H. Bart van der Worp, Johanna M. A. Visser-Meily, Rick M. Dijkhuizen

**Affiliations:** ^1^Biomedical MR Imaging and Spectroscopy Group, Center for Image Sciences, University Medical Center Utrecht and Utrecht University, Utrecht, Netherlands; ^2^Center of Excellence for Rehabilitation Medicine, UMC Utrecht Brain Center, University Medical Center Utrecht and Utrecht University, De Hoogstraat Rehabilitation, Utrecht, Netherlands; ^3^Department of Neurology and Neurosurgery, UMC Utrecht Brain Center, University Medical Center Utrecht and Utrecht University, Utrecht, Netherlands; ^4^Department of Rehabilitation, Physical Therapy Science and Sports, UMC Utrecht Brain Center, University Medical Center Utrecht and Utrecht University, Utrecht, Netherlands

**Keywords:** systematic review, meta-analysis, repetitive transcranial magnetic stimulation, motor function recovery, upper limb outcome, stroke

## Abstract

**Background:** Repetitive transcranial magnetic stimulation (rTMS) is a promising intervention to promote upper limb recovery after stroke. We aimed to identify differences in the efficacy of rTMS treatment on upper limb function depending on the onset time post-stroke.

**Methods:** We searched PubMed, Embase, and the Cochrane Library to identify relevant RCTs from their inception to February 2018. RCTs on the effects of rTMS on upper limb function in adult patients with stroke were included. Study quality and risk of bias were assessed independently by two authors. Meta-analyses were performed for outcomes on individual upper limb outcome measures (function or activity) and for function and activity measures jointly, categorized by timing of treatment initiation. Timing of treatment initiation post-stroke was categorized as follows: acute to early subacute (<1 month), early subacute (1–3 months), late subacute (3–6 months), and chronic (>6 months).

**Results:** We included 38 studies involving 1,074 stroke patients. Subgroup analysis demonstrated benefit of rTMS applied within the first month post-stroke [MD = 9.31; 95% confidence interval (6.27–12.34); *P* < 0.0001], but not in the early subacute phase (1–3 months post-stroke) [MD = 1.14; 95% confidence interval (−5.32 to 7.59), *P* = 0.73) or chronic phase (>6 months post-stroke) [MD = 1.79; 95% confidence interval (−2.00 to 5.59]; *P* = 0.35), when assessed with a function test [Fugl-Meyer Arm test (FMA)]. There were no studies within the late subacute phase (3–6 months post-stroke) that used the FMA. Tests at the level of function revealed improved upper limb function after rTMS [SMD = 0.43; 95% confidence interval (0.02–0.75); *P* = 0.0001], but tests at the level of activity did not, independent of rTMS onset post-stroke [SMD = 0.17; 95% confidence interval (−0.09 to 0.44); *P* = 0.19]. Heterogeneities in the results of the individual studies included in the main analyses were large, as suggested by funnel plot asymmetry.

**Conclusions:** Based on the FMA, rTMS seems more beneficial only when started in the first month post-stroke. Tests at the level of function are likely more sensitive to detect beneficial rTMS effects on upper limb function than tests at the level of activity. However, heterogeneities in treatment designs and outcomes are high. Future rTMS trials should include the FMA and work toward a core set of outcome measures.

## Introduction

In patients with stroke, paresis of the upper limb is a major cause of disability (1, 2). This motor disturbance influences activities of daily living, but also the quality of life of patients and their relatives (3, 4). Neurorehabilitation therefore often focuses on restoration of upper limb function. Several studies have suggested that non-invasive brain stimulation promotes recovery of the upper limb, possibly through enhancement of motor cortex plasticity (5, 6).

Repetitive transcranial magnetic stimulation (rTMS) is a non-invasive, painless method to modulate cortical excitability. High-frequency rTMS or intermittent theta-burst stimulation (TBS) can increase cortical excitability, whereas low-frequency rTMS or continuous TBS can suppress cortical excitability (7). Interhemispheric imbalance in primary motor cortex (M1) activity and the remaining functional motor output after stroke may contribute to motor dysfunction and has been suggested as target for therapeutic rTMS (8).

Earlier meta-analyses of small, randomized controlled trials (RCTs) suggest that rTMS is able to improve motor outcome in the paretic arm after stroke (9, 10). However, there are large differences between the results of RCTs, which could be explained by methodological differences (11, 12), including the timing of treatment initiation after stroke.

Research to date has not yet determined which time period post-stroke would be the optimal time window to start treatment. Many clinical practice guidelines advocate an early start of rehabilitation after stroke (13). Results from studies in animal models and patients suggest that there is an early critical time window during which the brain is most responsive to neurorehabilitation treatments (14). Most recovery takes place during the first 3 months, after which improvement is believed to reach a plateau phase (15, 16). However, it remains unknown whether rTMS interventions early after stroke could be more effective than at later points in time. Furthermore, the used outcome measure(s) to assess upper limb function must match with the stated intention of the treatment. Outcomes can be measured at the level of function, activity (capacity and performance) or participation, according to the International Classification of Function, Disability and Health (ICF model) (17). An outcome measure at function level [e.g., Fugl-Meyer Assessment (FMA)] may be more sensitive to effects of interventions targeted at the neural level, than outcome measures at the level of activity or participation [e.g., Action Research Arm Test (ARAT)], which are also affected by cognitive, personal and environmental factors (18).

We performed a systematic review and meta-analysis to evaluate whether the efficacy of rTMS on upper limb function depends on the time of treatment initiation after stroke. As secondary aims, we also assessed the efficacy of rTMS on upper limb function at the levels of function and activity (ICF model), and determined the efficacy of rTMS applied in the first month post-stroke on upper limb function assessed at 3 months post-stroke.

## Methods

This systematic review was conducted according to the Preferred Reporting Items for Systematic Reviews and Meta-Analyses (PRISMA) guidelines (19). We did not register the protocol in a registry prior to publishing.

### Search Strategy and Selection of Studies

We searched the literature in three databases (PubMed, Embase, Cochrane Library) for RCTs published up to February 2018 as a full-text article in the English language. We based our search on the following overarching PICO:

In adult patients (≥18 years) with stroke (population), does rTMS aimed at improvement of upper limb function (intervention) as compared with sham rTMS or no rTMS (comparison) improve function or activity of the upper limb (outcome)?

We used the key terms “stroke,” “transcranial magnetic stimulation,” “upper limb function,” or their synonyms (for a detailed search strategy, see Supplementary Table 1). Manual searches of the reference lists of the selected articles were also conducted.

Studies were excluded if rTMS was part of a coupling/priming protocol or if it was bilateral; if there was no upper limb outcome or stroke severity scale measurement (e.g., NIHSS score) as outcome assessment; or if information required to perform a meta-analysis (e.g., mean scores, standard deviations) was missing. When necessary, authors were contacted, or procedures were deployed for estimation of missing data (see Analyses). Two reviewers (EvL and RC) evaluated the retrieved literature based on titles and abstracts. Differences were discussed until consensus was reached.

### Critical Appraisal of Studies

The methodological quality and risk of bias of the included studies were evaluated with the PEDro scale (Physiotherapy Evidence Database from the Center for Evidence-Based Physiotherapy of The George Institute for Global Health) (20). The 11 items on the scale can be rated as present or absent, with a maximum score of 10 (one item is excluded in the PEDro score). The sum score was classified according to the Canadian Stroke Rehabilitation Evidence-Based Review (SREBR), which categorized the study quality as excellent (9–10), good (6–8), fair (4–5), or poor (0–3) (21). As a modification, studies scoring 6 or higher in which the critical criteria 2 or 3 (randomization and concealment of allocation, respectively) were absent, were downgraded to fair quality. The methodological quality and risk of bias were rated independently by two reviewers (EvL and RC), compared and discussed until consensus was reached.

### Data Extraction

The following data were extracted from the included studies: number of subjects; demographic and clinical characteristics of the subjects (age, gender, time since stroke); intervention protocols (type of rTMS and additional therapies, intensity, number of pulses and sessions, type of coil); outcome measures and mean differences and standard deviations (SDs) of the change scores or means and SDs of the scores after intervention. The extracted data was cross-checked by the second reviewer (RC).

We made an overview of all outcome measures used in the included studies and selected the outcome measures that were used in at least two studies, to enable analysis of results per individual outcome measure. Outcome measures were categorized according to the ICF model to group them at the level of function or activity for further analysis.

We made a categorization of time of treatment post-stroke according to the recent recommendations by the Stroke Recovery and Rehabilitation Roundtable (SRRR) taskforce (22): acute to early subacute (<1 month), early subacute (1–3 months), late subacute (3–6 months), and chronic (>6 months). The SRRR categorization of acute (1–7 days) and early subacute (7 days−3 months) were taken together and divided into acute to early subacute (<1 month) and early subacute (1–3 months), because most recovery of motor function takes place within the first 30 days post-stroke (23). In this way, all included studies could fit within a (specific) treatment timeframe. We checked whether the real and sham rTMS conditions of crossover studies could fit within the specific timeframe.

### Data Analysis

Cohen's kappa was calculated to check the interrater reliability of the selection and inclusion of articles.

For quantitative synthesis, effect sizes were calculated based on the change between baseline and post-intervention measurement, or the post-intervention score if the baseline score was not given, in the rTMS and control groups, divided by the pooled standard deviation. We calculated the standard deviation when only *t*-values and standard error of the mean (SEM) were reported. If there was no numerical data provided, we extracted these from the figures, using Plot Digitizer 2.6.8 based on the Cochrane Handbook for Systematic Reviews of Interventions (24). In case of repeated outcome assessments, the first assessment performed after the treatment was used to represent the post-intervention data. Crossover trials were included when point estimates and associated precision of point estimates were given, and when a washout period was incorporated. Standardized mean differences (SMDs), instead of the mean difference (MD), with 95% confidence intervals (95% CIs) were used if the outcome measurement scale was not identical between studies. The (unstandardized) mean difference was used when change-from-baseline scores were combined with post-intervention scores. If a study had multiple treatment groups, the results for the individual treatment conditions were compared. If the results were comparable, an overall effect for the different treatment conditions was computed and used (multiple comparison correction) (24).

To investigate the effect of rTMS treatment in the subacute or chronic phase after stroke, subgroup analyses were performed for the individual outcome measures found. Subgroup analyses of the different timings of post-stroke rTMS treatment were also performed for function and activity outcome measures jointly.

To determine the effect of rTMS applied within the first month post-stroke on upper limb function assessed 3 months post-stroke, an independent analysis was performed.

To determine the potential influence of rTMS frequency (low to the unaffected hemisphere vs. high to the affected hemisphere), number of treatment sessions and additional therapy (rTMS alone vs. rTMS + therapy), additional subgroup analyses were performed for the function and activity outcome measures (while maintaining the differentiation between subacute and chronic groups). At least two treatment time-points had to be represented in the subgroups, and a subgroup had to consist of at least one study.

The heterogeneity of the effect sizes was assessed with Cochran's *Q*-test and the inconsistency *I*^2^ index, in order to assess the consistency between the trials (24). The heterogeneity of the outcome measurements determined the use of a fixed or random effects method. When *I*^2^ was >50%, indicative of substantial heterogeneity, and the *P-*value from the Chi-squared test was below 0.05, a random effects model was applied. The weight of each study, for its effect on the pooled result, was determined by the sample size and confidence interval. The effect sizes were classified as small (<0.2), medium (0.2–0.8), or large (>0.8) (25).

A funnel plot was used to assess publication bias. Sensitivity analyses were conducted by omitting low quality studies (single-blind studies and without concealed treatment allocation) and studies with a crossover design to determine their influence on the effect size.

Analyses were performed with Review Manager, version 5.3 (26).

## Results

**Figure 1:**
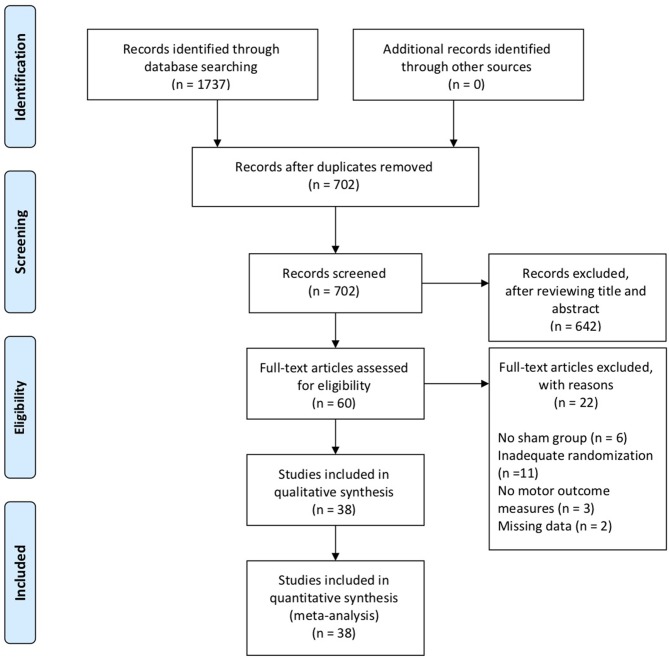
PRISMA flow chart.

Of 1,737 articles identified in the electronic database search, 38 were included in the systematic review, involving a total of 1,074 subjects. The interrater reliability, measured by Cohen's kappa, was 0.86, demonstrating almost perfect agreement (27). Figure 1 shows a flow diagram of the selection process.

### Characteristics of the Studies

Study characteristics are described in Table 1. All studies were designed as RCTs, and six of these studies were designed as randomized controlled crossover studies (28, 38, 46, 48, 52, 56). The real and sham rTMS conditions of the crossover studies took place within the specific timeframes (i.e., <1, 1–3, 3–6, >6 months post-stroke) and the wash-out periods ranged from 30 min to >1 week. The mean patient age in the studies ranged from 46 (52) to 75 (50) years. Nineteen studies (28–32, 34, 38–40, 45, 49, 51, 53, 55, 56, 59, 60, 62–64) included patients more than 6 months after stroke onset; six (33, 37, 41, 47, 52, 58) between 3 and 6 months after stroke, and 12 within 1 month after stroke. (35, 36, 42–44, 46, 48, 50, 54, 57, 65). The time between stroke onset and start of treatment varied from 6 days (36) to 4 years (39). Twenty-four studies were funded by grants from universities, governmental agencies, hospitals, or medical foundations. For 14 studies it was either explicitly mentioned that the work was not supported by any grant from the public or private sector or that there was nothing to disclose financially, or information on funding was not available.

**Table 1:** Characteristics of the included studies.

**References**	**No. of participants (Exp/Ctr)**	**Mean age (years) (Exp/Ctr)**	**Mean time Post-stroke**	**rTMS protocol**	Coil^*^/Location	**Control condition**	**Arm/hand outcome measurement**	**Additional intervention**
Ackerley et al. (28)	10 (crossover)	60	28 m	i/cTBS, 90% AMT, 600 stimuli, 1 session	UH, AH M1	Sham coil	ARAT	Upper-limb motor training
Ackerley et al. (29)	18 (9/9)	61/71	20/18 m	iTBS, 90% AMT, 600 pulses, 10 sessions	AH M1	Sham coil	ARAT, FMA	PT
Askin et al. (30)	40 (20/20)	58.8/56.75	28.35/24.35 m	1 Hz, 90% RMT, 1,200 pulses, 10 sessions	UH M1	Only PT	BRS, FMA, BBT, MAS, FIM, MMSE, FAS	PT
Avenanti et al. (31)	30 (8/8/14)	TMS-PT: 60.9/PT-TMS: 64.0/64.0	30.75/27.5/34.14 m	1 Hz, 90% RMT, 1,500 pulses, 10 sessions	UH M1	Tilted coil	JTT, NHPT, BBT	PT
Barros et al. (32)	20 (10/10)	57.4/64.6	9/8 m	1 Hz, 90% MT, 1,500 pulses, 10 sessions	UH M1	2 coils (1 connected and 1 disconnected)	MAS, FMA, FIM	PT
Cha et al. (33)	30 (15/15)	64.1/63.3	4.1/3.9 m	1 Hz, 90% RMT, 1,200 pulses, 20 sessions	Right hemisphere, P3 10/20 EEG system	Sham coil	Grip strength, BBT	Conventional rehabilitation
Chang et al. (34)	17 (9/8)	58.1/59.5	11.8/8.1 m	5 Hz, 80% RMT, 1,000 pulses, 10 sessions	AH M1	Tilted coil	JTT	Motor learning task
Conforto et al. (35)	30 (15/15)	54.8/56.7	27/28.3 d	1 Hz, 90% RMT, 1,500 pulses, 10 sessions	UH M1	Tilted coil	JTT, FMA, MRS	Conventional rehabilitation
Du et al. (36)	69 (23/23/23)	LF: 56.78/HF: 56.78/53.61	LF: 6/HF: 7/8 d	LF: 1 Hz, 110–120% RMT, HF: 3 Hz, 80–90% RMT, both: 1,200 pulses, 5 sessions	UH, AH M1	Tilted coil	FMA	Conventional rehabilitation
Emara et al. (37)	60 (20/20/20)	HF: 50.9/LF: 55/55.9	HF: 2.5/LF: 6.5/3.5 m	HF: 5 Hz, 80–90% MT, 750 pulses, LF: 1 Hz, 110–120% MT, 150 pulses, both10 sessions	UH, AH M1	Tilted coil	Finger tapping, MRS	PT
Etoh et al. (38)	18 (crossover)	59.7	29.9 m	1 Hz, 90% RMT, 240 pulses, 5 sessions	UH M1	Tilted coil	FMA, ARAT, STEF, MAS	PT
Fregni et al. (39)	15 (10/5)	57.7/52.6	3.52/3.97 y	1 Hz, 100% MT, 1,200 pulses, 5 sessions	UH M1	Sham coil	JTT, PTT, reaching time	–
Higgins et al. (40)	9 (4/5)	74/60	134/95 m	1 Hz, 110% RMT, 1,200 pulses, 8 sessions	UH M1	Sham coil	BBT, WMFT, MAL, grip and pinch strength	Functional task practice
Hosomi et al. (41)	39 (18/21)	62.4/63.2	46.1/45.1 d	5 Hz, 90% RMT, 500 pulses, 10 sessions	AH M1	Tilted coil	BRS, FMA, FIM, NIHSS, hand grip, finger tapping	Conventional rehabilitation
Hsu et al. (42)	12 (6/6)	56.8/62.3	22.0/20.8 d	iTBS, 5 Hz, 80% AMT, 1,200 pulses, 10 sessions	AH M1	Tilted coil	FMA, ARAT	Conventional rehabilitation
Khedr et al. (43)	36 (12/12/12)	LF: 54.7 HF: 59.0/60.0	LF: 16.3 HF: 17.2/17.7 d	LF: 1 Hz, 100% RMT, 900 pulses HF: 3 Hz, 130% RMT, 900 pulses, both: 5 sessions	UH, AH M1	Tilted coil	Strength hand grip, Keyboard tapping, PPT	Conventional rehabilitation
Khedr et al. (44)	48 (16/16/16)	3 Hz: 58.25/10 Hz: 58.37/58	3 Hz: 8, 10 Hz: 6/6.2 m	(1): 3 Hz, 130% RMT, (2): 10 Hz, 100% RMT, both: 750 pulses, 5 sessions	AH M1	Tilted coil	Strength hand grip/shoulder abduction/hip flexion	Conventional rehabilitation, medical treatment
Lai et al. (45)	38 (21/17)	62.6/62.1	10.4/10.6 m	iTBS, 80% AMT, 600 pulses, 10 sessions	AH M1	Sham coil	WMFT, finger tapping	PT
Liepert et al. (46)	12 (crossover)	63	7.3 d	1 Hz, 90% RMT, 1,200 pulses, 1 session	UH M1	Sham coil	Grip strength, NHPT	–
Lüdemann-Podubecka et al. (47)	40 (20/20)	68.3/65.7	1.6/1.7 m	1 Hz, 100% RMT, 900 pulses, 15 sessions	UH M1	0% RMT	WMFT, MESUPES, finger tapping	Functional task practice
Lüdemann-Podubecka et al. (48)	10 (crossover)	71.9	1 m	1 Hz, 110% RMT, 900 pulses, 1 session	UH M1	0% RMT	JTT, BBT	–
Malcolm et al. (49)	19 (9/10)	68.4/65.7	3.9/3.8 y	20 Hz, 90% MT, 2,000 pulses, 10 sessions	AH M1	Sham coil	WMFT, MAL, BBT	Conventional rehabilitation
Matsuura et al. (50)	20 (10/10)	72.2/74.7	9.4/9.8 d	1 Hz, 100% RMT, 1,200 pulses, 5 sessions	UH M1	Tilted coil	FMA, PPT, grip strength	–
Motamed Vaziri et al. (51)	12 (6/6)	55.17/57	24/23 m	1 Hz, 60–80% MT, 1,200 pulses, 10 sessions	UH M1	ns	BRS, FMA, grip strength	Conventional rehabilitation
Nowak et al. (52)	15 (crossover)	46	1.93 m	1 Hz, 100% RMT, 600 pulses, 1 session	UH M1	Vertex	Finger tapping, reach to grasp	–
Özkeskin et al. (53)	21 (10/11)	55.7/64.5	10.5/24.5 m	1 Hz, 90% RMT, 1,500 pulses, 10 sessions	UH M1	Sham coil	FMA, JTT, BRS	Hand manipulation + UL training
Pomeroy et al. (54)	24 (realreal: 6/realsham: 4/shamreal: 7/shamsham: 7)	69.2/64.8/78.9/81	25.2/34/27.3/26.9 d	1 Hz, 1.2 × MT, 200 pulses, 8 sessions	AH M1	Sham coil	ARAT	Voluntary muscle contraction
Rose et al. (55)	19 (9/10)	64.7/64.6	60.4/62.8 m	1 Hz, 100% RMT, 1,200 pulses, 16 sessions	UH M1	Sham coil	WMFT, ARAT, FMA, MAL, MAS	Functional task practice
Sankarasubramanian et al. (56)	15 (crossover)	62.13	57 m	M1: 1 Hz, PMd: 5 Hz, both: 90% AMT, 1,500 pulses, 1 session	UH M1, UH PMd	Tilted coil	Reaching time	–
Sasaki et al. (57)	29 (HF: 9/LF: 11/9)	HF: 65.7/LF: 68.6/63.0	HF: 18.4/LF: 17.0/15.4 d	HF: 10 Hz,1,000 pulses, LF: 1 Hz, 1,800 pulses, both: 90% RMT, 5 sessions	AH, UH M1	Tilted coil	BRS, grip strength, tapping frequency	–
Seniow et al. (58)	40 (20/20)	63.5/63.4	41.7/38.0 d	1 Hz, 90% RMT, 1,800 pulses, 15 sessions	UH M1	Sham coil	WMFT, FMA	PT
Takeuchi et al. (59)	20 (10/10)	58.4/59.6	25.2/28.7 m	1 Hz, 90% RMT, 1,500 pulses, 1 session	UH M1	Tilted coil	Pinch force	Motor training
Takeuchi et al. (60)	20 (10/10)	61.2/63.4	25.4/34.4 m	1 Hz, 90% RMT, 1,500 pulses, 1 session	UH M1	Tilted coil	Pinch force and acceleration	Motor training
Tosun et al. (61)	25 (9/7/9)	57.6/56/61.3	49.3/59.6/47.2 d	1 Hz, 90% RMT, 1,200 pulses, 10 sessions	UH M1	Only PT	FMA	EMG-FNMS
Theilig et al. (62)	24 (12/12)	61.8/60.2	8.6/2.7 m	1 Hz, 100% RMT, 900 pulses, 10 sessions	UH M1	0% RMT	WMFT	EMG-FNMS
Vongvaivanichakul et al. (63)	14(7/7)	56.9/58.7	27.7/58.3 m	1 Hz, 90% RMT, 1,200 pulses, 1 session	UH M1	Tilted coil	WMFT, reach to grasp	Reach-to-grasp training
Wang et al. (64)	44 (16/14/14)	cM1: 62.38/cPMd 63.07/68	6/9/7 m	cM1/cPMd: 1 Hz, 600 pulses, 90% RMT, 20 sessions	UH M1/PMd	Sham coil	FMA, WMFT, MRC	Conventional rehabilitation
Zheng et al. (65)	112 (58/54)	65.4/66.2	19.3/18.7 d	1 Hz, 90% RMT, 1,800 pulses, 24 sessions	UH M1	Sham coil	FMA, WMFT	Virtual reality

RMT, resting motor threshold; PT, physiotherapy; EMG-FNMS, electromyography-triggered functional neuromuscular stimulation; UH, unaffected hemisphere; M1, primary motor cortex; cM1, contralesional M1; cPMd, contralesional dorsal premotor cortex; Hz, hertz; m, months; d, days; y, years; Exp, experimental group; Ctr, control group; BRS, Brunnstrom Recovery Stages; FMA, Fugl-Meyer; BBT, Box and Block Test; MAS, Modified Asworth Scale; FIM, Functional Independence Measure; MMSE, Mini-Mental State Examination; FAS, Functional Ability Scale; NIHSS, National Institutes of Health Stroke Scale; JTT, Jebsen Taylor Test; ARAT, Action Research Arm Test; PPT, Purdue Pegboard Test; MRS, Modified Rankin Scale; WMFT, Wolf Motor Function Test; MESUPES, Motor Evaluation Scale for Upper Extremity in Stroke Patients; MAL, Motor Activity Log; NHPT, Nine Hole Peg Test; STEF, Simple Test for Evaluating Hand Function; MRC, Medical Research Council; ns, not specified;

* *All studies used a figure of eight coil; MAS, Modified Ashworth Scale*.

### Treatment Characteristics

Different TMS treatment protocols were used in the included studies. In 25 studies, 1 Hz rTMS was applied to the unaffected hemisphere, with 200–1,800 pulses per session (30–33, 35, 38–40, 46–48, 50–55, 58–60, 62–66). Three studies applied high-frequency rTMS to the affected hemisphere with frequencies ranging from 5 (500 and 1,000 pulses) to 20 Hz (2,000 pulses) (34, 41, 49). Intermittent TBS to the affected hemisphere with 600 or 1,200 pulses was applied in three studies (29, 42, 45). Seven studies applied a combination of low- and high-frequency rTMS (ranging from 1 to 10 Hz) or a combination of continuous and intermittent TBS (ranging from 150 to 1,800 pulses) to the unaffected or affected hemispheres, respectively [crossover study design (26, 38), low/high or continous/intermittent group and sham group (33, 35, 47, 48, 54)].

In all studies the primary motor cortex was targeted, of which two studies also targeted the premotor cortex (56, 64). One study was an exception, as only the P3 area (based on a 10/20 EEG system) was targeted (33).

All studies used a figure-of-eight coil for real rTMS treatment. Sham stimulations were executed with sham coils, tilted coils or real coils without stimulator output, or by vertex stimulation. One study did not describe details of the sham rTMS (51). The treatment protocol period ranged from 1 session (28, 46, 48, 52, 56, 59, 60, 63) to 24 sessions (65).

Ten different additional therapies were used in combination with the rTMS protocol. The program of the therapy was not always defined, and conventional rehabilitation differed between studies, e.g., conventional rehabilitation could consist of physical therapy and occupational therapy, but could also involve functional task practice or passive limb movement. Conventional rehabilitation (eleven studies) and physical therapy (eight studies) were the most frequently applied additional therapeutic interventions. Virtual reality training, reach to grasp training, a motor learning task and voluntary muscle contraction were used in the other studies. For the studies with an outcome assessment 3 months post-stroke, it was unclear if patients received the additional therapy (i.e., physical-, conventional-, and physiotherapy) also after rTMS. Seven studies did not report or included a therapeutic intervention in addition to rTMS.

### Outcome Measurements

The arm/hand motor scales on which outcomes were assessed varied across the studies, and some studies used multiple outcome measures. For meta-analysis, eight different arm/hand motor scales were selected and classified as measures of (body) function: Fugl-Meyer Arm (FMA), Reaching Time (RT), Grip Strength (GS), Tapping Frequency (TF), and Pinch Force (PF), or as measures of activity: Jebsen Taylor Test (JTT), Wolf Motor Function Test (WMFT), and Action Research Arm Test (ARAT). Measures of (body) function signify measures of motor impairment.

The FMA was the most frequently used test in the included studies (*n* = 16), of which five used this as the primary outcome measure (30, 32, 36, 51, 61). The other outcome measures were less frequently used and a minority of the studies (*n* = 6) included the WMFT or TF (Table 1) (40, 52, 56, 59, 60, 63). All studies assessed the scales mentioned above before and after treatment. More than half of the included studies (*n* = 21) had only a single post-intervention measurement. The remaining studies included outcome measurements at multiple time-points, up to 1 year (44) after the intervention.

### Methodological Quality and Risk of Bias

Total scores on the PEDro scale for the included studies ranged from 4 (51) to 10 (29, 32, 33, 36, 37, 40, 47, 55, 58, 65). There were no low quality studies (PEDro score ≤ 3). Eligibility criteria, random allocation, between-group statistical comparisons, point estimates and measures of variability were reported in all studies. Seventeen (45%) studies did not report if treatment allocation was concealed, and in another 17 studies the treating therapists were not blinded (Table 2).

**Table 2:** Assessment of risk of bias of the included studies.

**References**	**Criteria**											**Total (max. score 10)**	**Quality**
	**1**	**2**	**3**	**4**	**5**	**6**	**7**	**8**	**9**	**10**	**11**		
Ackerley et al. (28)	Y	1	0	1	1	1	1	1	1	1	1	9	Fair
Ackerley et al. (29)	Y	1	1	1	1	1	1	1	1	1	1	10	Excellent
Askin et al. (30)	Y	1	1	1	0	0	1	1	1	1	1	8	Good
Avenanti et al. (31)	Y	1	1	1	1	1	1	1	0	1	1	9	Excellent
Barros et al. (32)	Y	1	1	1	1	1	1	1	1	1	1	10	Excellent
Cha et al. (33)	Y	1	1	1	1	0	1	1	1	1	1	10	Excellent
Chang et al. (34)	Y	1	0	1	1	0	0	1	1	1	1	8	Fair
Conforto et al. (35)	Y	1	1	1	1	1	0	1	1	1	1	9	Excellent
Du et al. (36)	Y	1	1	1	1	1	1	1	1	1	1	10	Excellent
Emara et al. (37)	Y	1	1	1	1	1	1	1	1	1	1	10	Excellent
Etoh et al. (38)	Y	1	0	0	1	1	1	1	1	1	1	8	Fair
Fregni et al. (39)	Y	1	0	1	1	1	1	1	1	1	1	9	Fair
Higgins et al. (40)	Y	1	1	1	1	1	1	1	1	1	1	10	Excellent
Hosomi et al. (41)	Y	1	1	1	1	0	1	1	1	1	1	9	Excellent
Hsu et al. (42)	Y	1	0	1	1	1	1	1	1	1	1	9	Fair
Khedr et al. (43)	Y	1	1	1	1	1	1	1	1	1	1	9	Excellent
Khedr et al. (44)	Y	1	1	1	1	0	1	0	1	1	1	8	Good
Lai et al. (45)	Y	1	0	1	1	1	0	1	1	1	1	8	Fair
Liepert et al. (46)	Y	1	0	0	1	0	1	0	0	1	1	5	Fair
Lüdemann-Podubecka et al. (47)	Y	1	1	1	1	1	1	1	1	1	1	10	Excellent
Lüdemann-Podubecka et al. (48)	Y	1	1	1	1	0	1	0	0	1	1	7	Good
Malcolm et al. (49)	Y	1	0	1	1	1	1	1	1	1	1	9	Fair
Matsuura et al. (50)	Y	1	0	1	1	0	1	1	1	1	1	8	Fair
Motamed Vaziri et al. (51)	Y	1	0	1	0	0	0	0	0	1	1	4	Fair
Nowak et al. (52)	Y	1	0	1	0	0	0	1	1	1	1	6	Fair
Özkeskin et al. (53)	Y	1	1	0	1	0	1	1	1	1	1	8	Good
Pomeroy et al. (54)	Y	1	1	0	1	1	1	1	1	1	1	9	Excellent
Rose et al. (55)	Y	1	1	1	1	1	1	1	1	1	1	10	Excellent
Sankarasubramanian et al. (56)	Y	1	0	0	1	0	0	1	1	1	1	6	Fair
Sasaki et al. (57)	Y	1	0	1	1	0	1	1	1	1	1	8	Fair
Seniow et al. (58)	Y	1	1	1	1	1	1	1	1	1	1	10	Excellent
Takeuchi et al. (59)	Y	1	0	1	1	1	1	0	0	1	1	7	Fair
Takeuchi et al. (60)	Y	1	0	1	1	1	1	0	0	1	1	7	Fair
Tosun et al. (61)	Y	1	0	1	0	0	1	1	0	1	1	6	Fair
Theilig et al. (62)	Y	1	1	0	1	0	1	0	0	1	1	6	Good
Vongvaivanichakul et al. (63)	Y	1	0	0	0	0	0	1	1	1	1	5	Fair
Wang et al. (64)	Y	1	1	1	1	1	0	1	1	1	1	9	Excellent
Zheng et al. (65)	Y	1	1	1	1	0	1	1	1	1	1	10	Excellent

*Criteria numbers: 1, eligibility criteria; 2, random allocation; 3, concealed allocation; 4, similar groups at baseline; 5, blinding subjects; 6, blinding therapists; 7, blinding assessors; 8, outcome obtained in more than 85% of the subjects; 9, intention-to-treat analysis; 10, between-group statistical comparisons; 11, point estimates and measures of variability*.

### Meta-Analysis

#### Subacute vs. Chronic Treatment

**Figure 2:**
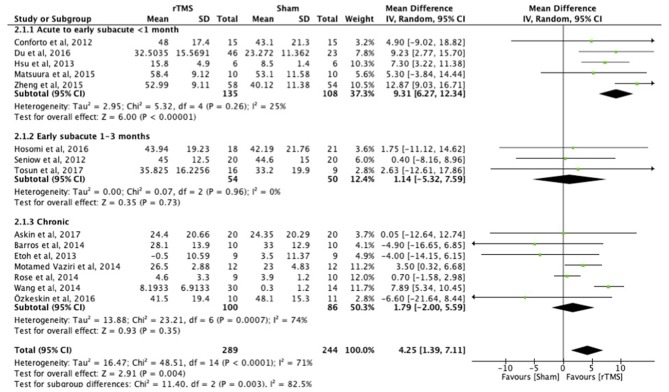
Effects of rTMS on the FMA scale, comparing different treatment onset times. Estimates of effect size are shown with 95% CIs. Final value and change scores combined as mean differences. The mean difference (MD) and 95% confidence intervals (CIs); No studies within 3–6 months post-stroke subgroup.

When assessed with the FMA, the benefit of early treatment (<1 month post-stroke) was larger than that of treatment in the early subacute phase (1–3 months) and chronic phase (Figure 2). Separate analyses indicated the difference between the different post-stroke phases. The acute to early subacute phase (<1 month) explicitly compared to the early subacute (1–3 months) phase showed a significant subgroup difference in favor of the acute to early subacute phase (*p* = 0.02). The acute to early subacute phase also showed a benefit when compared to the chronic phase (*p* = 0.002) (Supplementary Figures 1–3 in Supplementary Table 2). For the other scales, ICF function and activity measures, the effects of early and late treatment did not differ (Supplementary Figures 1–8 in Supplementary Table 3). However, the ICF function measures RT and FT (Supplementary Figures 1, 2 in Supplementary Table 3) did show an overall positive effect of rTMS on upper limb function for the early treatment group (FT), which was not observed when treatment was started later. Sensitivity analysis showed minimal impact on the results after removal of the crossover, single-blind and no treatment allocation studies (Supplementary Table 4). The funnel plot showed that the estimated treatment effects scattered around the total overall estimate of the meta-analysis (Supplementary Table 5). Asymmetry in the funnel plot is noticeable.

#### Function vs. Activity

**Figure 3:**
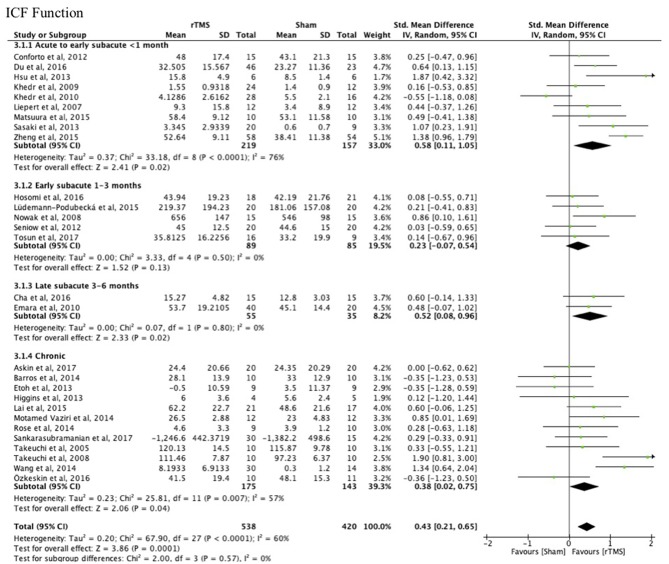
Effects of rTMS on the ICF Function domain, comparing different treatment onset times. Estimates of effect size are shown with 95% CIs. The standardized mean difference (SMD) and 95% confidence intervals (CIs); ICF Function measures: Fugl-Meyer Arm, Grip Strength, Finger Tapping, and Pinch Force.

**Figure 4:**
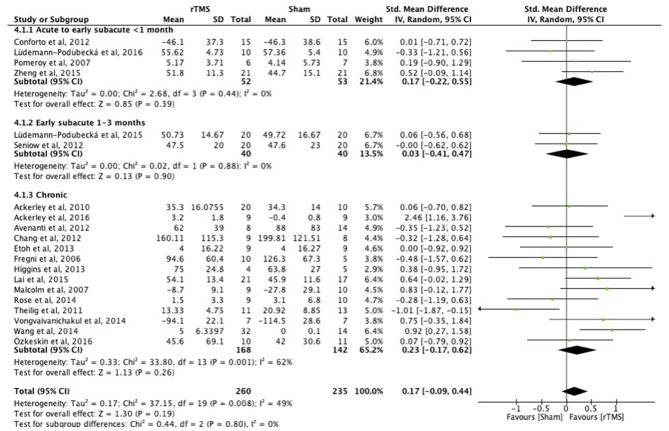
Effects of rTMS on the ICF Activity domain, comparing different treatment onset times. Estimates of effect size are shown with 95% CIs. The standardized mean difference (SMD) and 95% confidence intervals (CIs); No studies within 3–6 months post-stroke subgroup; ICF Activity measures: Jebsen Taylor Test, Action Research Arm Test, and Wolf Motor Function Test.

There were no differences between the early and late treatment groups for studies categorized as assessing ICF function (FMA, GS, FT, and PF) and activity (JTT, ARAT, and WMFT) measures (Figures 3, 4). A benefit of real rTMS was only observed when outcomes were assessed with an ICF function measure (Figure 3). Sensitivity analysis showed minimal impact on the results after removal of the crossover, single-blind, and no treatment allocation studies (Supplementary Table 4).

### Treatment Within 1 Month and Outcome at 3 Months

**Figure 5:**
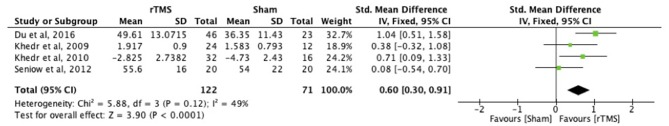
Effects of rTMS applied within 1 month with outcome assessment at 3 months post-stroke. Estimates of effect size are shown with 95% CIs. The standardized mean difference (SMD) and 95% confidence intervals (CIs); ICF Function measures: Fugl-Meyer Arm and Grip Strength.

rTMS intervention within 1 month after stroke improved upper limb function at 3 months (*p* < 0.0001; Figure 5) (ICF function measures: FMA and GS).

### Other Subgroup Analyses

Subgroup analyses (number of treatment sessions, additional therapy, rTMS frequency/site of stimulation) revealed statistically significant beneficial treatment effects on the ICF function measures, but not on the ICF activity measures (Supplementary Table 6). In the analysis in which the number of treatment sessions was divided into different subgroups (1 session, 2–10 sessions, and 11–20 sessions), improved upper limb function was found for all the different number of treatment sessions on the ICF function measures (all *p* < 0.05) (Supplementary Figures 1–5 in Supplementary Table 6). Subgroup analysis of rTMS alone and rTMS combined with additional therapy showed significant effects on upper limb ICF function measures for both rTMS treatment approaches (Supplementary Figures 6, 7 in Supplementary Table 6). In another subgroup analysis, significant mean effect sizes were found for both low- and high frequency rTMS (to the unaffected and affected hemispheres, respectively) (Supplementary Figures 10, 11 in Supplementary Table 6). In all the subgroup analyses no significant differences were found between different rTMS post-stroke onset times.

## Discussion

This systematic review and meta-analysis demonstrate that rTMS within 1 month after stroke leads to greater improvement on the FMA than rTMS applied after 1–3 months or after 6 months. In addition, independent from treatment onset time, rTMS seems to have a positive effect on upper limb function if assessed with tests that targeted (body) function specifically, which was not evident with tests assessing activity. Lastly, when rTMS treatment was started in the first month after stroke, upper limb function was still improved at 3 months after stroke, the time of outcome assessment in most acute stroke trials.

### Timing of rTMS Treatment Onset After Stroke

The beneficial effect of rTMS, applied within 1 month after stroke, on the FMA score have not been previously described. In an earlier systematic review and meta-analysis of rTMS after stroke, which also evaluated the arm/hand motor scales separately for upper limb function, no effect of rTMS followed by upper limb training on motor outcome measures, including the FMA, was found (11). However, this systematic review included only eight studies and patients treated within 1 month after stroke were excluded. A recent randomized sham-controlled trial (*n* = 199) that was published after the search period of our meta-analysis found no difference between active and sham rTMS treatment groups, combined with motor training, on the FMA (nor on the ARAT or WMFT). This lack of difference may be attributed to the inclusion of patients beyond 3 months after stroke (67). The results from our meta-analysis also differ from findings from a recent meta-analysis of transcranial direct current stimulation treatments after stroke, in which increased capacity of activities of daily living (ADL), but not increased arm function, measured by the FMA, were reported after tDCS (68). A reason for the discrepancy between these results and our findings may be the difference in included post-stroke time points (ranging between 3 days up to 8 years post-stroke for the meta-analysis of tDCS treatments). The discrepancy could also be attributed to the different mechanisms underlying cortical excitability changes after rTMS and tDCS. TMS can directly induce action potentials, whereas tDCS does not evoke action potentials but modifies neuronal membrane polarization (7, 69). This can result in different neuromodulatory responses between rTMS and tDCS stimulated neural networks. Another explanation might be that the improvement in ADL capacity is not a reflection of improvement in arm function but of generalized treatment effects. In addition, patients with a non-functional arm may be independent in ADL (70).

### Body Function Measures

The FMA, a measure of body function, has recently been recommended as a primary outcome measure for intervention trials targeting the upper limb throughout different phases after stroke (71, 72). While rTMS improved FMA scores specifically when applied in the first month, this effect was not observed for other body function measures. This may at least partly be explained by the higher number of studies that assessed upper limb function with the FMA (*n* = 15) than with the other body function measures (RT, FT, GS, and PF) (*n* ≤ 8). Consequently, the low numbers of patients (sample sizes: 6–60) in studies that used other measures and no power calculations may have led to insufficient power to detect differences. Another possible explanation may be that the FMA assesses multiple components of the upper limb, such as the shoulder, elbow, wrist, hand, fingers, and coordination, and is based on the different sequential stages of motor recovery. According to the FMA stages of recovery (based on the Brunnstrom Approach), basic synergy patterns appear in one of the first stages, and points can be awarded in each stage (73). By contrast, other body function measures assess or focus on fine motor control, and points are only awarded when the patient can move freely from the synergy pattern. Consequently, some patients will not be able to perform the fine motor tasks assessed with these scales and possible improvements in distal musculature cannot be captured. However, if rTMS treatment started in the chronic phase post-stroke and outcome was assessed with measures at ICF function level, other than the FMA, these patients displayed a favorable response to the treatment. It is possible that these patients developed compensatory movements to accomplish the function tests, e.g., by using additional trunk movements (74, 75).

### Comparisons to Previous Studies

Two earlier meta-analyses also performed a subgroup analysis for rTMS effects at different times after stroke (10, 76). These analyses also showed more pronounced effects of rTMS applied in the (sub)acute phase (2 weeks to 6 months) than in the chronic phase (>6 months) post-stroke. However, these meta-analyses pooled studies with outcome measures at different levels of ICF (i.e., function and activity), which increases methodological variation. Furthermore, not all findings were corrected for multiple comparisons (76) and few studies selectively included patients at specific post-stroke stages (10).

Earlier meta-analyses have considered the potential influence of rTMS frequency/site of stimulation (10, 76), number of sessions (76) and upper-limb training (11) on upper limb function. Two meta-analyses revealed more pronounced effects on upper limb function following low-frequency rTMS to the unaffected hemisphere as compared to high-frequency rTMS to the affected hemisphere (10, 76). Low-frequency rTMS protocols have been more frequently used than high-frequency protocols to promote upper limb recovery, throughout the different post-stroke phases. In the current meta-analysis, both the low- and high-frequency studies revealed significant effects on upper limb function measured by ICF function measures. Outcome measures have not previously been categorized according to their measurement level (ICF) in meta-analyses. Prior studies had shown that five rTMS sessions have the most beneficial effects on upper limb function compared to a single session or more than 10 sessions (76). In contrast to these findings, our subgroup analyses showed that there were significant beneficial effects on ICF function measures for varying amount of treatment sessions (i.e., single treatment session, 2–10 or 11–20 sessions), however this finding is based on few studies within the different phases of treatment onset post-stroke. Regarding additional therapy next to rTMS treatment, one study did not find support that the combination of rTMS with upper-limb training would be more beneficial on upper limb function than upper-limb training alone (11). In our analysis, additional therapy combined with rTMS was found to have a similar effect as rTMS alone. However, the effect of specific types or intensity of additional therapy, paired with rTMS, has not been investigated yet.

### Outcome Measure Selection

To effectively capture the multidimensional aspects of post-stroke dysfunction and recovery, it has been recommended to measure outcome at different levels of function, activity and participation (ICF model) (71). Outcome measures at the level of function are more directly linked to stroke-related brain changes as compared to outcome measures at the level of activity, which are also strongly affected by cognitive, environmental and personal factors (18, 77). This could explain why we found no effect of rTMS treatment on activity outcome measures. High heterogeneity and wide confidence intervals of effect sizes were found for some analyses on activity outcome measures, which could also account for the absence of rTMS effects in activity.

It is important that the selected outcome measures within a trial reflect the underlying rationale or mechanism of the intervention under study. Furthermore, interventions targeted at one or more specific parts of the upper limb (i.e., arm, hand, shoulder) should select an outcome measure that is capable of specifically assessing effects on those parts or subtest scores of an outcome measure should be reported to indicate at what level of the upper limb the most significant effects occur. However, for several tests it is not entirely clear to which ICF domain they belong. For example, some outcome measures at activity level (e.g., ARAT and WMFT) also contain a number of test items at function level and vice versa. Effects of interventions which directly influence neural activity, such as rTMS, are probably best assessed with outcome measures that are able to capture the neural recovery process. For motor function, this may be achieved with the FMA. In addition, inclusion of arm/hand motor scales at the level of activity and participation as secondary outcomes can be valuable to evaluate if treatment effects generalize to daily life. Objective kinematic measurements may offer a valuable addition to the existing and widely used outcome measures. These quantitative assessments can provide more detailed insights into key components of motor recovery, such as individual finger movements, smoothness of reaching, force control, and trunk displacement (78, 79). A combination of outcome measures at different ICF domains, including the use of kinematic measures, can also prevent a patient from becoming discouraged if the performance on a particular test fails.

### Study Strengths and Limitations

The beneficial effect of rTMS applied in the acute to early subacute phase post-stroke is in agreement with theories on a critical time window post-stroke for obtaining recovery-enhancing effects (14). Our review showed that when rTMS was applied in the first month after stroke, a beneficial effect on upper limb function could still be measured at 3 months post-stroke. A three-month post-stroke assessment has been recommended by the Stroke Recovery and Rehabilitation Roundtable for stroke recovery trials, especially when interventions target neural repair processes, which may be most prevalent during this timeframe (71). In addition, assessment after 6 months can inform on outcome at a stage when spontaneous recovery often reaches a plateau, particularly in more severe strokes (80).

There are limitations in our review and meta-analysis that need to be reported. Firstly, since our study was dependent on the type and quality of the data in the individual studies, risks of bias that could lead to inflation of the effect size estimates should be acknowledged. Therefore, the results need to be interpreted with caution. There were some examples of risk of bias. In some subgroup analyses only one study was representative of a subgroup. Heterogeneities in the results of the individual studies included in the main analyses were large, as suggested by funnel plot asymmetry. Measurement of effect sizes of treatment was often based on a mixture of change scores and final scores. However, unpublished studies with negative findings may have been missed due to publication bias, may also have led to funnel plot asymmetry. The methodological quality of the studies was fair to excellent, but almost half of the studies were single-blind and did not conceal the treatment allocation or describe the allocation procedure. Nevertheless, our sensitivity analyses showed no significant changes in results when those studies were excluded. Also, we might have missed relevant studies published in non-English languages. Another potential source of bias in clinical research is the type of funding or sponsorship. Although none of the studies were funded by an industrial partner, bias can also result from non-commercial funding sources with specific interests. Secondly, because of the large variations in the study populations, we could not examine possibly confounding effects of differences in demographic and stroke-related characteristics between the studies. Age, gender, level of cognition, depression, severity of impairment and physical activity are examples of confounders that could influence motor performance. Thirdly, due to the limited data we could not adequately account for differences in rTMS protocols and frequencies/sites of stimulation, experimental designs, additional therapy, motor scores (e.g., FMA subscores, clinical vs. kinematic measures), and patient inclusion criteria. We focused on effects of rTMS applied at different times post-stroke, whereby investigating the role of (intensity of) additional therapy such as virtual reality therapy and functional task practice, and single rTMS sessions could not be performed.

## Conclusions

rTMS treatment within the first month after stroke seems more beneficial in increasing upper limb function than after 1–3 months or in the chronic phase post-stroke (>6 months). Improvements after rTMS can most likely be detected with outcome measures assessing body functions, like the FMA score, than tests at the level of activity (e.g., JTT, ARAT). However, rTMS treatment studies in stroke patients are highly heterogeneous, with varying outcome measures and relatively small sample sizes. Another source of uncertainty is that we are unable to identify whether improved outcomes were primarily caused by rTMS *per se* or by rTMS in combination with an additional therapy (of a certain intensity). Further research and international cooperation should be undertaken to develop a standardized, core set of measurements for testing upper limb function. We recommend to conduct measurements at the different levels of function, activity (and participation). Future studies should incorporate these standardized tests, include a follow-up measurement at 3 months after stroke onset (if the trial starts within 1 month post-stroke), and report their findings in a uniform manner (e.g., using final scores or change scores, and subtest scores).

## Data Availability Statement

The datasets generated for this study are available on request to the corresponding author.

## Author Contributions

EL and RD designed this study. EL performed data extraction and statistical analysis. EL, HW, JV-M, and RD revised and approved the final manuscript.

### Conflict of Interest

The authors declare that the research was conducted in the absence of any commercial or financial relationships that could be construed as a potential conflict of interest.
