# Supplementary material for: Timing of Repetitive Transcranial Magnetic Stimulation Onset for Upper Limb Function After Stroke: A Systematic Review and Meta-Analysis
Source: Front Neurol. 2019 Dec 3;10:1269. doi: 10.3389/fneur.2019.01269 (PMC6901630; doi:10.3389/fneur.2019.01269)
Supplement: Supplementary file 1 [file Table_1.DOCX]

**Embase**‘cerebrovascular accident’/exp

‘brain ischemia’/exp

‘brain hemorrhage’/exp

‘cerebrovascular accident’ OR ‘cerebral ischemia’ OR ‘cerebral ischaemia’ OR ‘brain ischemia’ OR ‘brain ischaemia’ OR ‘brain infarction’ OR ‘intracranial hemorrhage’ OR ‘intracranial haemorrhage’ OR ‘intracerebral hemorrhage’ OR ‘intracerebral haemorrhage’ OR ‘intracranial embolism’ OR ‘intracranial thrombus’ OR ‘lacunar infarct’ OR ‘lacunar stroke’ OR ‘post stroke’ OR ‘poststroke’ OR ‘brain vascular accident’ OR ‘cerebral infarct’ OR ‘brain hemorrhage’ OR ‘brain haemorrhage’

AND

‘transcranial magnetic stimulation’/exp
‘Transcranial Magnetic’ OR ‘repetitive transcranial’ OR ‘rTMS’ OR ‘TBS’ OR ‘theta burst’

AND

‘upper limb’/exp OR
‘upper limb function’ OR ‘upper extremity’ OR ‘upper extremities’ OR ‘arm’ OR ‘arms’ OR ‘hand’ OR ‘hands’ or ‘paresis’ OR ‘pareses’

**Pubmed**

(("Transcranial Magnetic Stimulation"[Mesh] OR
Transcranial Magnetic[Title/Abstract] OR
repetitive transcranial[Title/Abstract] OR
rTMS[Title/Abstract] OR
TBS[Title/Abstract] OR
theta burst[Title/Abstract] OR
paired pulse magnet*[Title/Abstract] OR
paired pulse tms[Title/Abstract] OR
paired associative[Title/Abstract])

AND

("Stroke"[Mesh] OR "Brain Ischemia"[Mesh] OR
"Intracranial Hemorrhages"[Mesh] OR
stroke[Title/Abstract] OR
CVA[Title/Abstract] OR
CVAs[Title/Abstract] OR
cerebrovascular accident*[Title/Abstract] OR
cerebral ischemia*[Title/Abstract] OR
cerebral ischaemia*[Title/Abstract] OR
brain ischemia*[Title/Abstract] OR
brain ischaemia*[Title/Abstract] OR
brain infarction*[Title/Abstract] OR
intracranial hemorrhage*[Title/Abstract] OR
intracranial haemorrhage*[Title/Abstract] OR
intracerebral hemorrhage*[Title/Abstract] OR
intracerebral haemorrhage*[Title/Abstract] OR
intracranial embolism*[Title/Abstract] OR
intracranial thromb*[Title/Abstract] OR
lacunar infarct*[Title/Abstract] OR
lacunar stroke*[Title/Abstract] OR
poststroke[Title/Abstract] OR
post stroke[Title/Abstract] OR
brain vascular accident*[Title/Abstract] OR
cerebral infarct*[Title/Abstract] OR
brain hemorrhage*[Title/Abstract] OR
brain haemorrhage*[Title/Abstract]))

AND

("Upper Extremity"[Mesh] OR
upper extremit*[Title/Abstract] OR
arm[Title/Abstract] OR arms[Title/Abstract] OR
hand[Title/Abstract] OR hands[Title/Abstract] OR
"Paresis"[Mesh] OR paresis[Title/Abstract] OR
pareses[Title/Abstract])

**Cochrane Library**

cerebrovascular accident or cerebral ischemia or cerebral ischaemia or brain ischemia or brain ischaemia or brain infarction or intracranial hemorrhage or intracranial haemorrhage or intracerebral hemorrhage or intracerebral haemorrhage or intracranial embolism or intracranial thrombus or lacunar infarct or lacunar stroke or post stroke or poststroke or brain vascular accident or cerebral infarct or brain hemorrhage or brain haemorrhage

Transcranial Magnetic or repetitive transcranial or rTMS or TBS or theta burst

Upper Extremity or upper extremit* or arm or arms or hand or hands or Paresis or paresis or pareses or upper limb*
