# Supplementary material for: Timing of Repetitive Transcranial Magnetic Stimulation Onset for Upper Limb Function After Stroke: A Systematic Review and Meta-Analysis
Source: Front Neurol. 2019 Dec 3;10:1269. doi: 10.3389/fneur.2019.01269 (PMC6901630; doi:10.3389/fneur.2019.01269)
Supplement: Supplementary file 2 [file Table_2.DOCX]

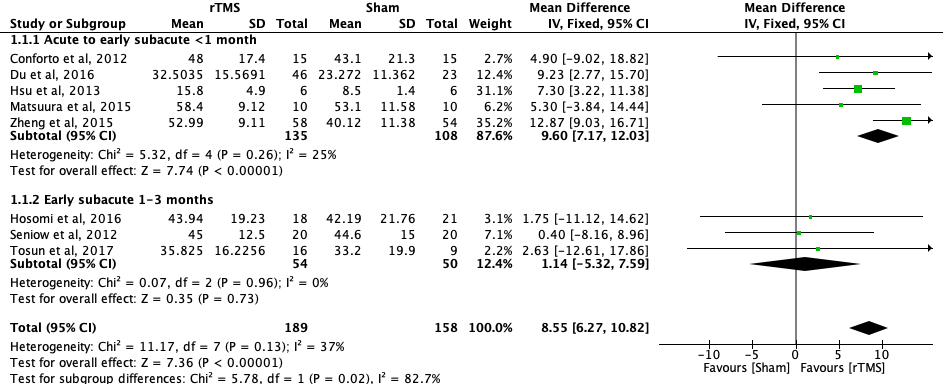


The mean difference (MD) and 95% confidence intervals (CIs)

**Supplementary Figure 1.** Effects of rTMS on the FMA scale, comparing the acute to early subacute (<1 month) and early subacute (1-3 months) treatment onset times. Estimates of effect size are shown with 95% CIs.


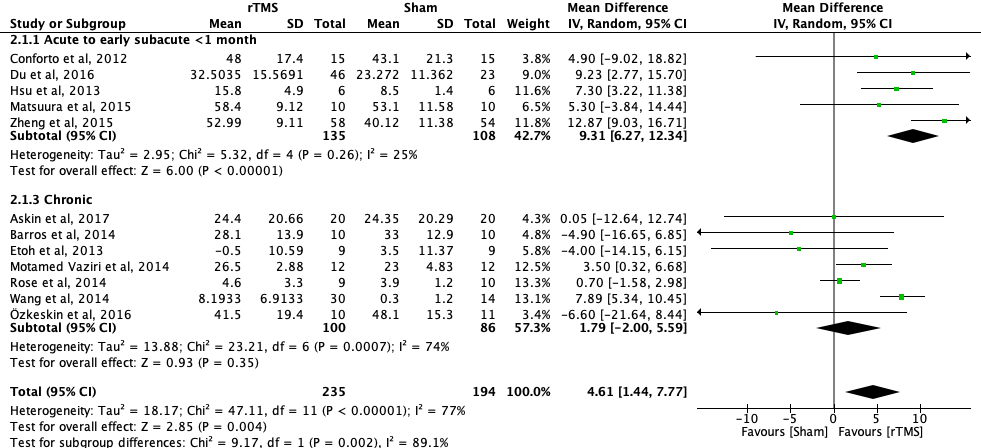


The mean difference (MD) and 95% confidence intervals (CIs)

**Supplementary Figure 2.** Effects of rTMS on the FMA scale, comparing the acute to early subacute (<1 month) and chronic (>6 months) treatment onset times. Estimates of effect size are shown with 95% CIs.


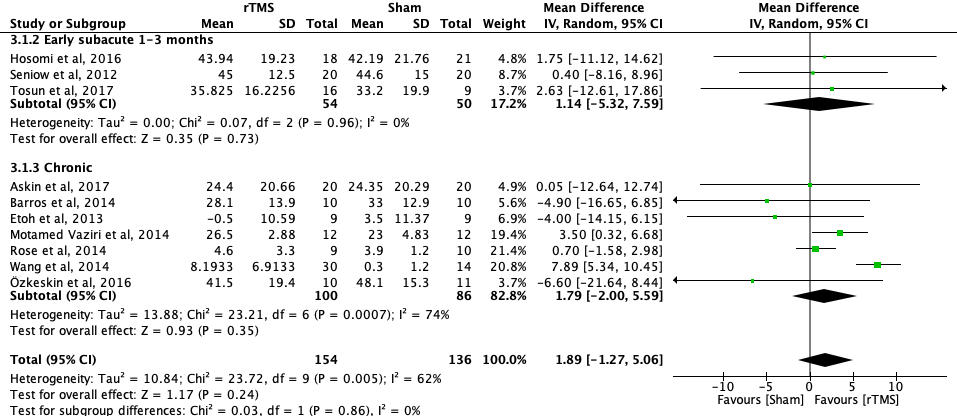


The mean difference (MD) and 95% confidence intervals (CIs)
**Supplementary Figure 3.** Effects of rTMS on the FMA scale, comparing the early subacute (1-3 months) and chronic (>6 months) treatment onset times. Estimates of effect size are shown with 95% CIs.
