# Supplementary material for: Timing of Repetitive Transcranial Magnetic Stimulation Onset for Upper Limb Function After Stroke: A Systematic Review and Meta-Analysis
Source: Front Neurol. 2019 Dec 3;10:1269. doi: 10.3389/fneur.2019.01269 (PMC6901630; doi:10.3389/fneur.2019.01269)
Supplement: Supplementary file 3 [file Table_3.DOCX]

Reaching Time (RT)


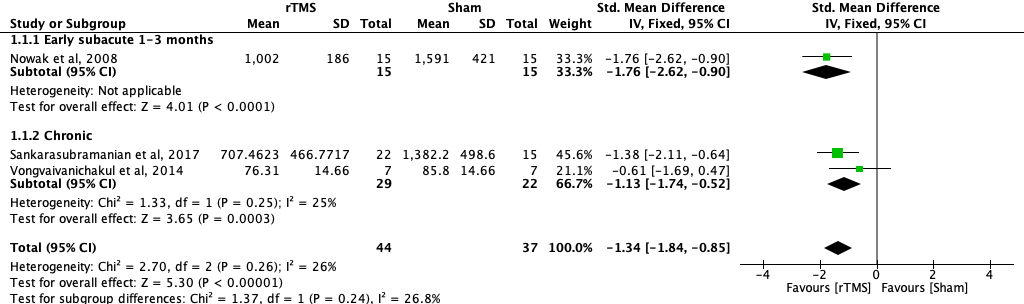
The standardized mean difference (SMD) and 95% confidence intervals (CIs); No studies within <1 month and 3-6 months post-stroke subgroups.
**Supplementary Figure 1.** Effects of rTMS on the RT scale, comparing different treatment onset times. Estimates of effect size are shown with 95% CIs.

Finger Tapping (FT)


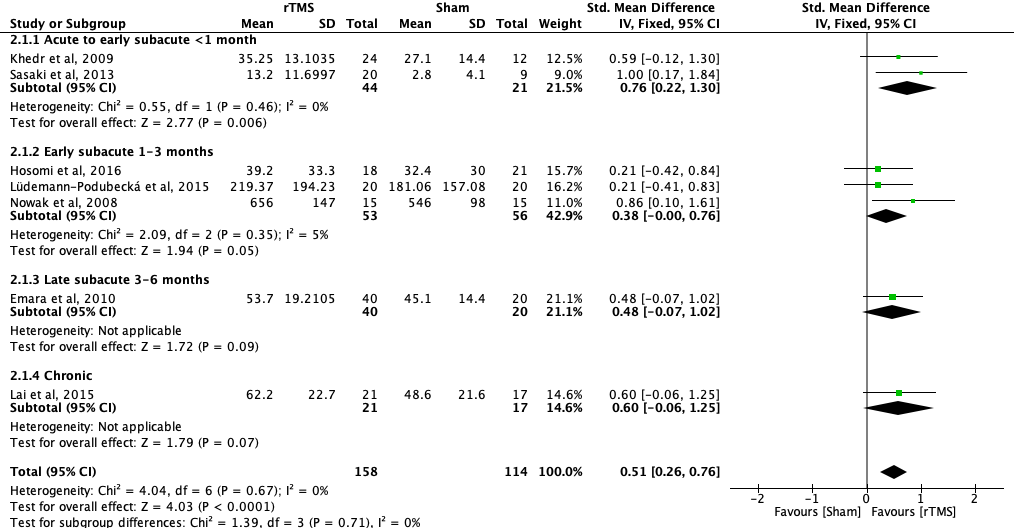


The standardized mean difference (SMD) and 95% confidence intervals (CIs)
**Supplementary Figure 2.** Effects of rTMS on the FT scale, comparing different treatment onset times. Estimates of effect size are shown with 95% CIs.

Wolf Motor Function Test (WMFT)
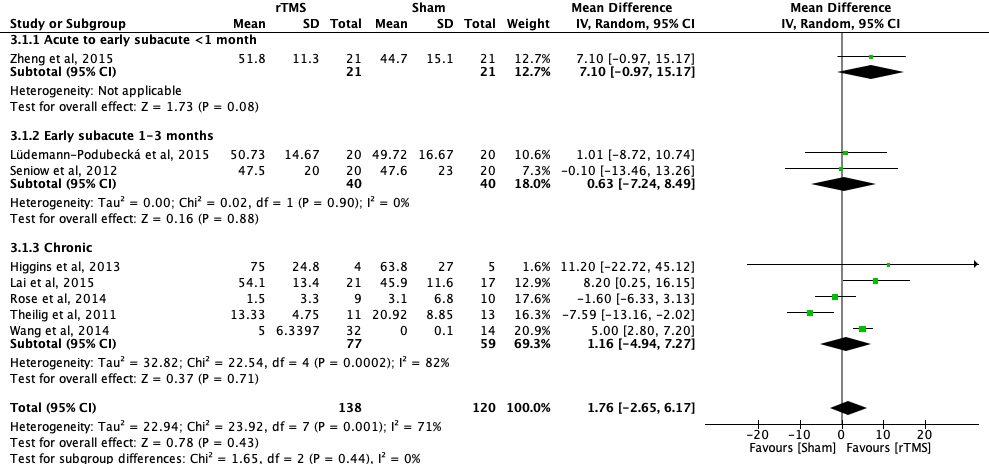
The mean difference (MD) and 95% confidence intervals (CIs); No studies within 3-6 months post-stroke subgroup.
**Supplementary Figure 3.** Effects of rTMS on the WMFT scale, comparing different treatment onset times. Estimates of effect size are shown with 95% CIs. Final value and change scores combined as mean differences.

Wolf Motor Function Test in seconds (WMFT-sec)


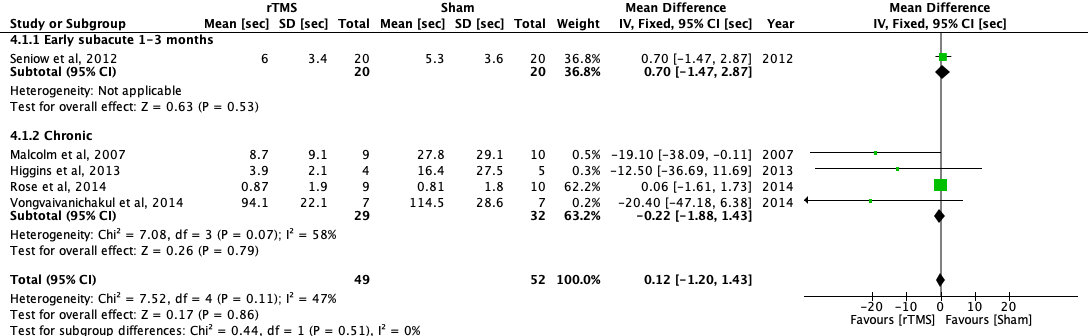


The mean difference (MD) and 95% confidence intervals (CIs); No studies within <1 month and 3-6 months post-stroke subgroups.
**Supplementary Figure 4.** Effects of rTMS on the WMFT-sec scale, comparing different treatment onset times. Estimates of effect size are shown with 95% CIs.

Grip Strength (GS)


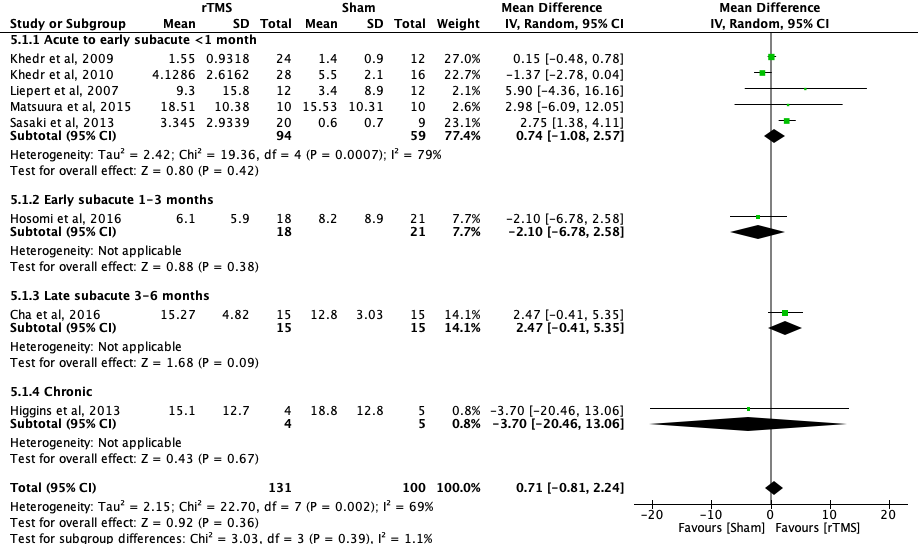


The mean difference (MD) and 95% confidence intervals (CIs)
**Supplementary Figure 5.** Effects of rTMS on the GS scale, comparing different treatment onset times. Estimates of effect size are shown with 95% CIs.

Action Research Arm Test (ARAT)


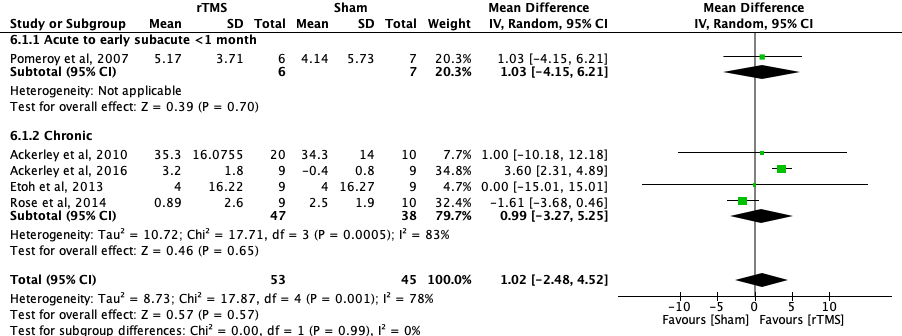


The mean difference (MD) and 95% confidence intervals (CIs); No studies within 1-3 and 3-6 months post-stroke subgroups.
**Supplementary Figure 6.** Effects of rTMS on the ARAT scale, comparing different treatment onset times. Estimates of effect size are shown with 95% CIs.

Jebsen Taylor Test (JTT)


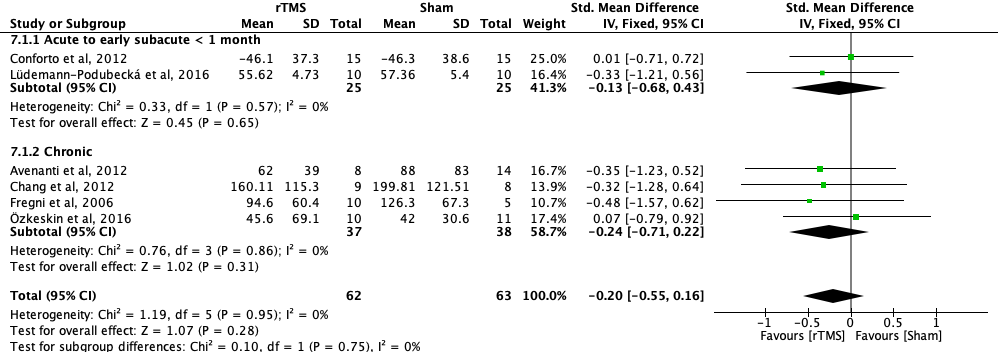
The standardized mean difference (SMD) and 95% confidence intervals (CIs); No studies within 1-3 and 3-6 months post-stroke subgroups.

**Supplementary Figure 7.** Effects of rTMS on the JTT scale, comparing different treatment onset times. Estimates of effect size are shown with 95% CIs.

Pinch Force (PF)


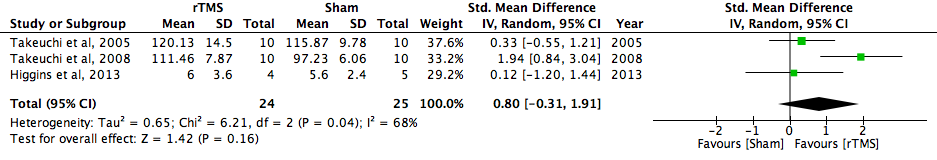
The standardized mean difference (SMD) and 95% confidence intervals (CIs)
**Supplementary Figure 8.** Effects of rTMS on the PF scale. Estimates of effect size are shown with 95% CIs.
