# Supplementary material for: Timing of Repetitive Transcranial Magnetic Stimulation Onset for Upper Limb Function After Stroke: A Systematic Review and Meta-Analysis
Source: Front Neurol. 2019 Dec 3;10:1269. doi: 10.3389/fneur.2019.01269 (PMC6901630; doi:10.3389/fneur.2019.01269)
Supplement: Supplementary file 4 [file Table_4.DOCX]

Fugl-Meyer Arm (FMA)


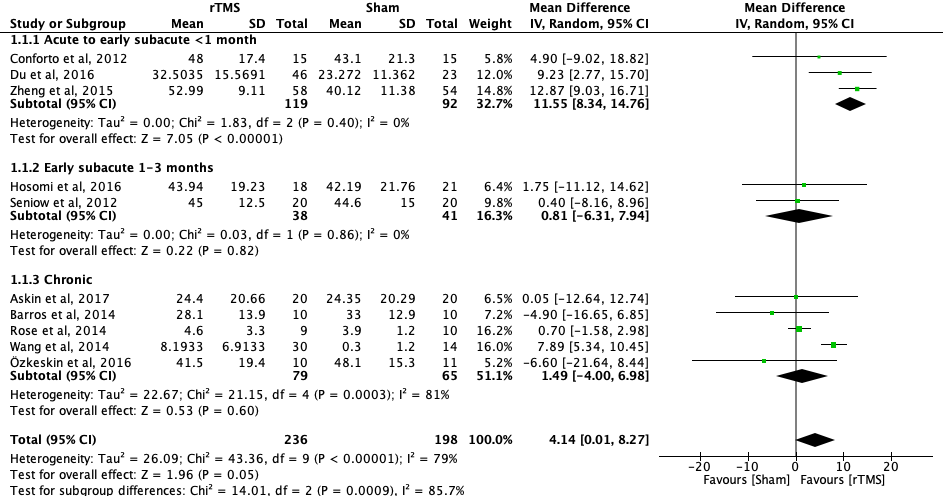


The mean difference (MD) and 95% confidence intervals (CIs); No studies within 3-6 months post-stroke subgroup.
**Supplementary Figure 1.** Effects of rTMS on the FMA scale without crossover, single-blind and no treatment allocation studies, comparing different treatment onset times. Estimates of effect size are shown with 95% CIs. Final value and change scores combined as mean differences.

Finger Tapping (FT)


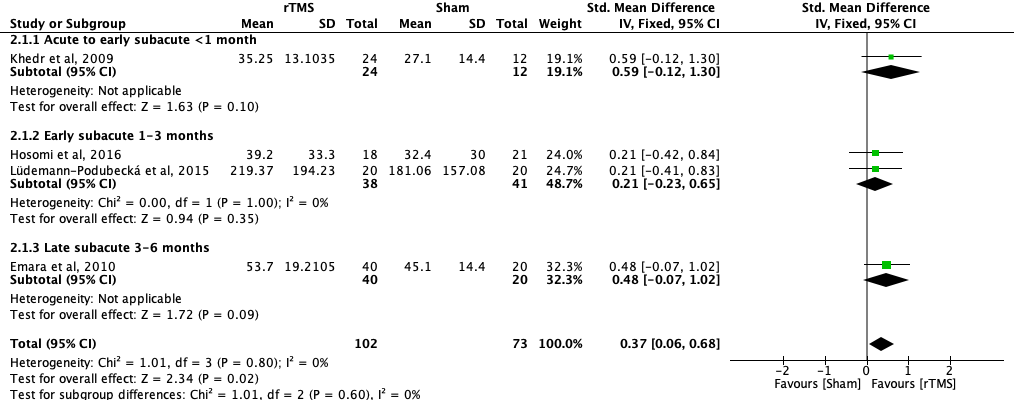
The standardized mean difference (SMD) and 95% confidence intervals (CIs); No studies within >6 months post-stroke subgroup.

**Supplementary Figure 2.** Effects of rTMS on the FT scale without crossover, single-blind and no treatment allocation studies, comparing different treatment onset times. Estimates of effect size are shown with 95% CIs.

Wolf Motor Function Test (WMFT)


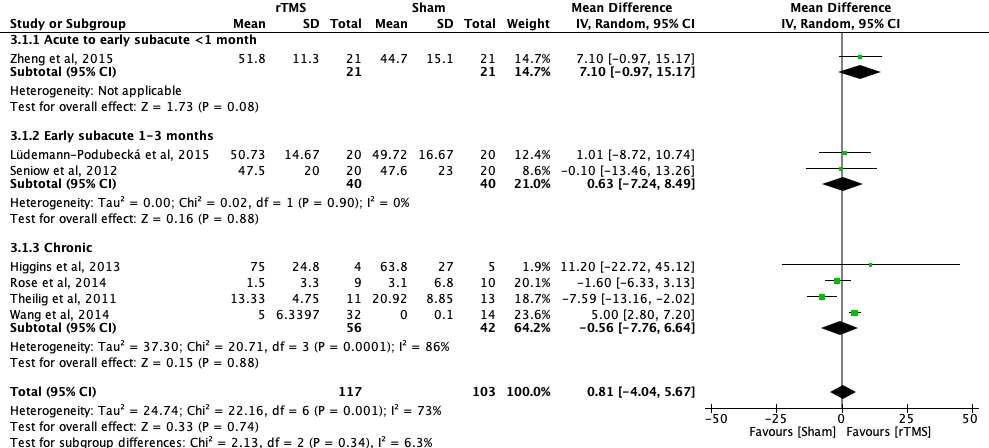

The mean difference (MD) and 95% confidence intervals (CIs); No studies within 3-6 months post-stroke subgroup.
**Supplementary Figure 3.** Effects of rTMS on the WMFT scale without crossover, single-blind and no treatment allocation studies, comparing different treatment onset times. Estimates of effect size are shown with 95% CIs. Final value and change scores combined as mean differences.

Wolf Motor Function Test in seconds (WMFT-sec)


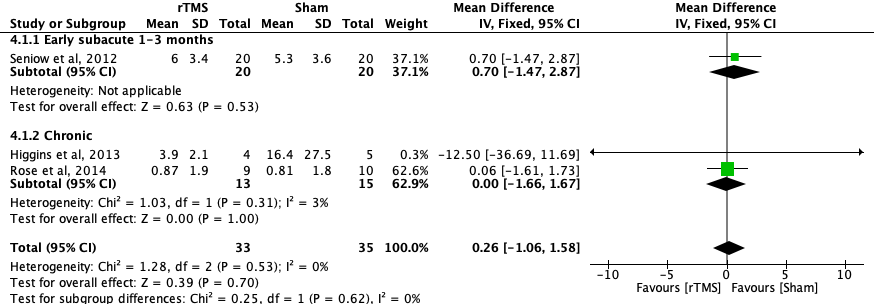

The mean difference (MD) and 95% confidence intervals (CIs); No studies within <1 month and 3-6 months post-stroke subgroups.

**Supplementary Figure 4.** Effects of rTMS on the WMFT-sec scale without single-blind and no treatment allocation studies, comparing different treatment onset times. Estimates of effect size are shown with 95% CIs. Final value and change scores combined as mean differences.

Grip Strength (GS)


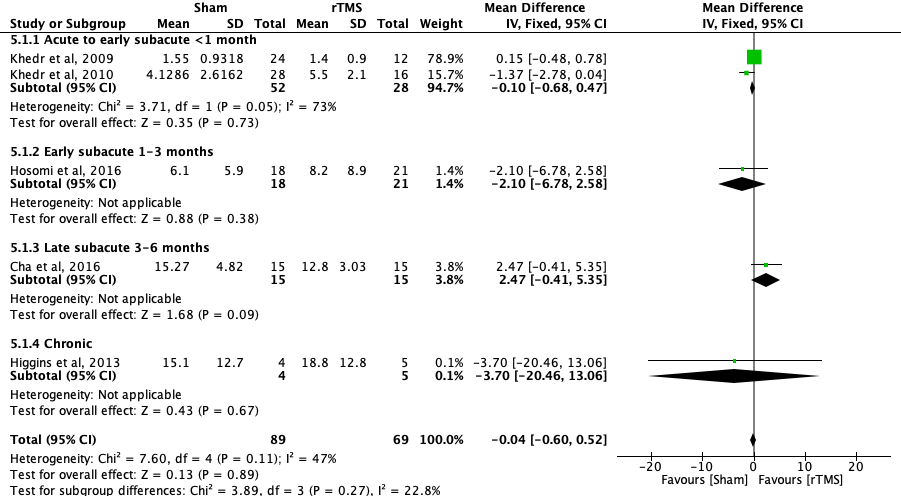

The mean difference (MD) and 95% confidence intervals (CIs)
**Supplementary Figure 5.** Effects of rTMS on the GS scale without single-blind and no treatment allocation studies, comparing different treatment onset times. Estimates of effect size are shown with 95% CIs. Final value and change scores combined as mean differences.

Action Research Arm Test (ARAT)


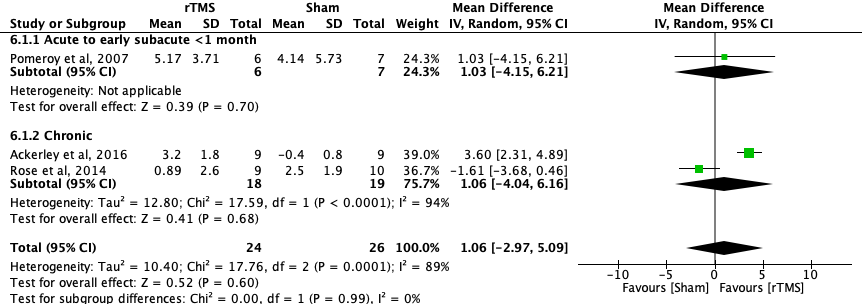

The mean difference (MD) and 95% confidence intervals (CIs); No studies within 1-3 and 3-6 months post-stroke subgroups.
**Supplementary Figure 6.** Effects of rTMS on the ARAT scale without single-blind and no treatment allocation studies, comparing different treatment onset times. Estimates of effect size are shown with 95% CIs.

Jebsen Taylor Test (JTT)


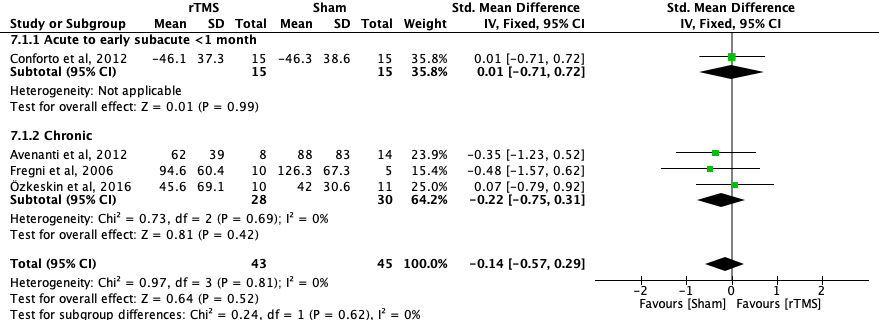

The standardized mean difference (SMD) and 95% confidence intervals (CIs); No studies within 1-3 and 3-6 months post-stroke subgroups.
**Supplementary Figure 7.** Effects of rTMS on the JTT scale without single-blind and no treatment allocation studies, comparing different treatment onset times. Estimates of effect size are shown with 95% CIs.

ICF Function


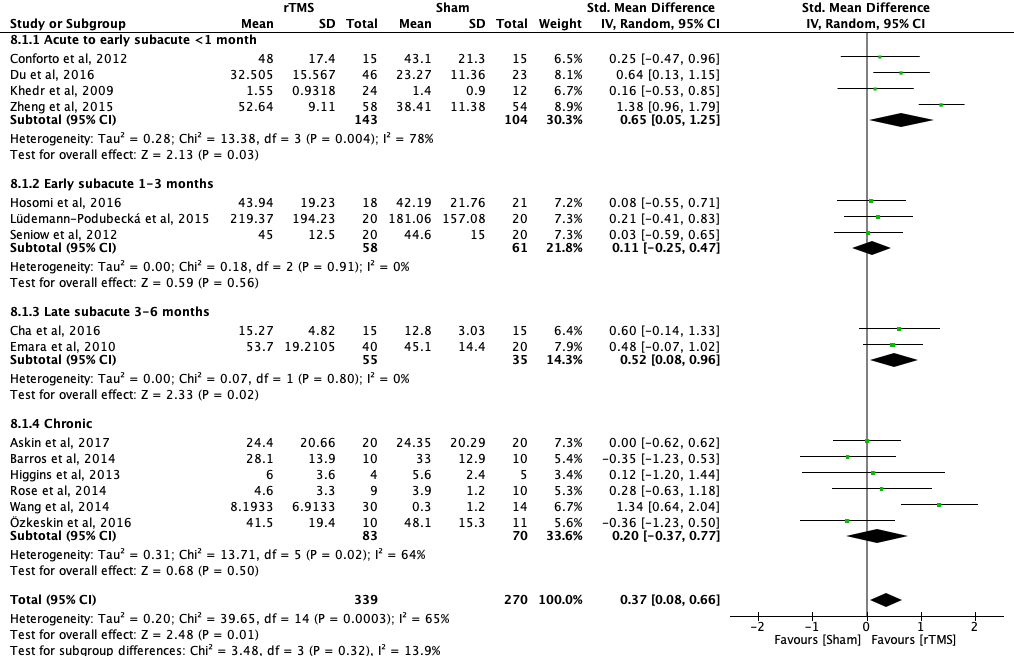

The standardized mean difference (SMD) and 95% confidence intervals (CIs)
**Supplementary Figure 8.** Effects of rTMS on the ICF Function domain without single-blind and no treatment allocation studies, comparing different treatment onset times. Estimates of effect size are shown with 95% CIs.

ICF Activity


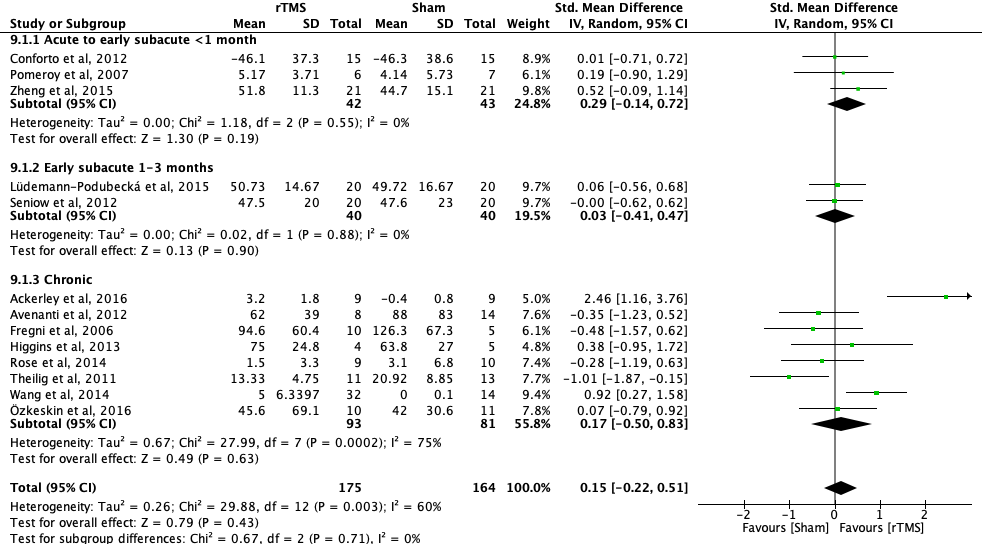

The standardized mean difference (SMD) and 95% confidence intervals (CIs); No studies within 3-6 months post-stroke subgroup.

**Supplementary Figure 9.** Effects of rTMS on the ICF Activity domain without single-blind and no treatment allocation studies, comparing different treatment onset times. Estimates of effect size are shown with 95% CIs.
