# Supplementary figures and images for: Timing of Repetitive Transcranial Magnetic Stimulation Onset for Upper Limb Function After Stroke: A Systematic Review and Meta-Analysis
Source: Front Neurol. 2019 Dec 3;10:1269. doi: 10.3389/fneur.2019.01269 (PMC6901630; doi:10.3389/fneur.2019.01269)

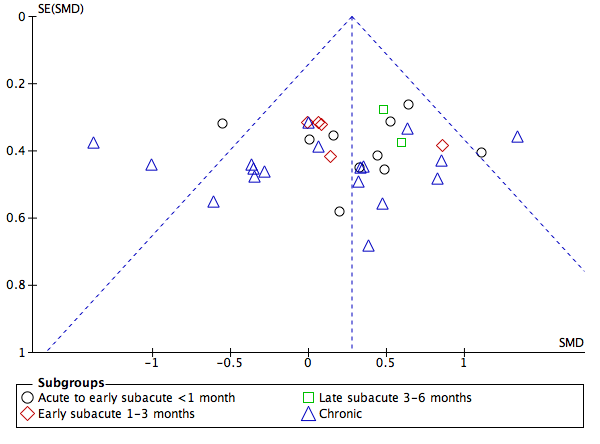


**Supplementary Figure 1**. Funnel plot

Supplement: Supplementary file 5 [file Table_5.DOCX]
