# Supplementary material for: Timing of Repetitive Transcranial Magnetic Stimulation Onset for Upper Limb Function After Stroke: A Systematic Review and Meta-Analysis
Source: Front Neurol. 2019 Dec 3;10:1269. doi: 10.3389/fneur.2019.01269 (PMC6901630; doi:10.3389/fneur.2019.01269)
Supplement: Supplementary file 6 [file Table_6.DOCX]

**A** Number of treatment sessions

ICF Function (1 session)


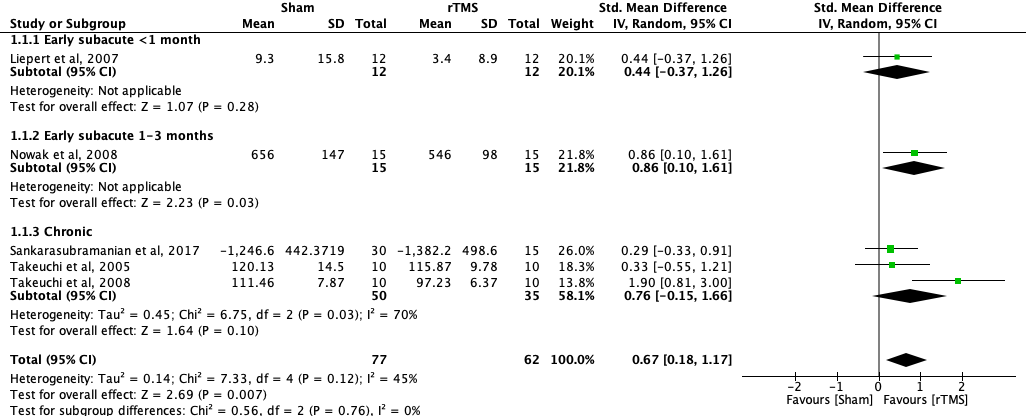
The standardized mean difference (SMD) and 95% confidence intervals (CIs); No studies within 3-6 months post-stroke subgroup.
**Supplementary Figure 1.** Effects of rTMS on the ICF Function domain with 1 treatment session, comparing different treatment onset times. Estimates of effect size are shown with 95% CIs.

ICF Activity (1 session)

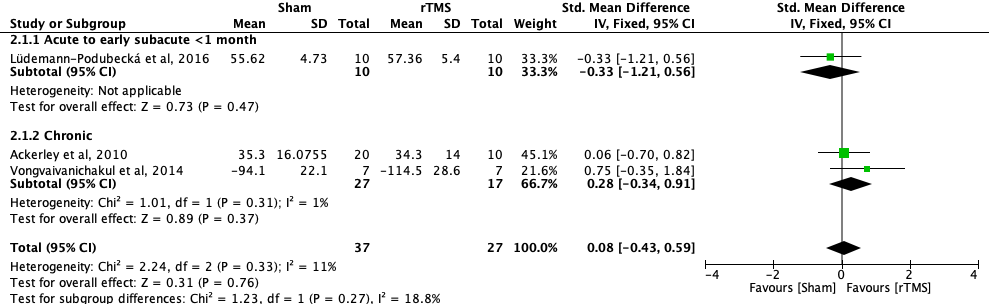

The standardized mean difference (SMD) and 95% confidence intervals (CIs); No studies within 1-3 and 3-6 months post-stroke subgroups.
**Supplementary Figure 2.** Effects of rTMS on the ICF Activity domain with 1 treatment session, comparing different treatment onset times. Estimates of effect size are shown with 95% CIs.

ICF Function (2-10 sessions)
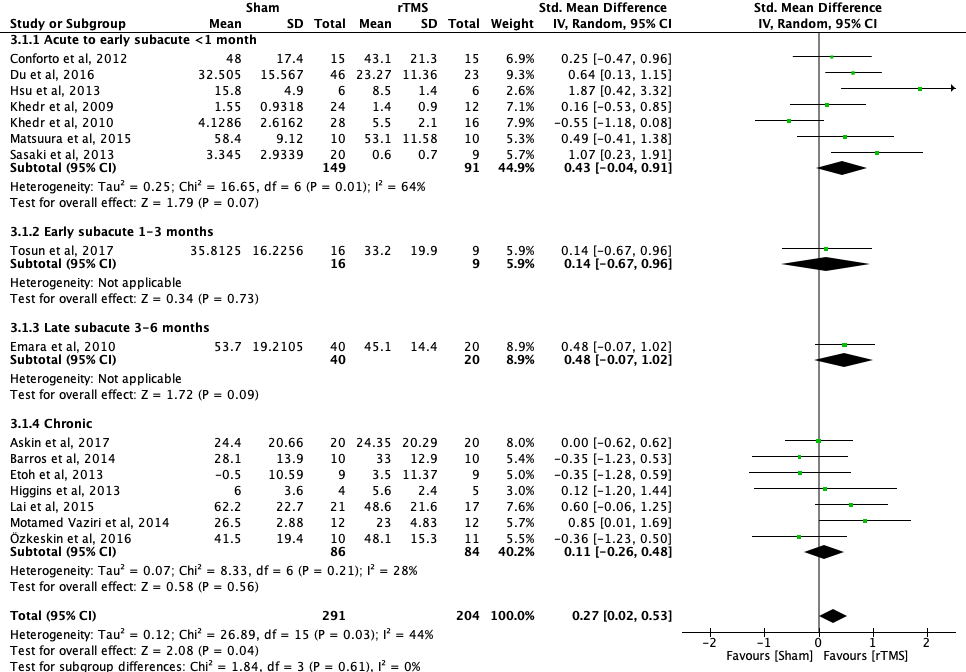

The standardized mean difference (SMD) and 95% confidence intervals (CIs)
**Supplementary Figure 3.** Effects of rTMS on the ICF Function domain with 2-10 treatment sessions, comparing different treatment onset times. Estimates of effect size are shown with 95% CIs.

ICF Activity (2-10 sessions)
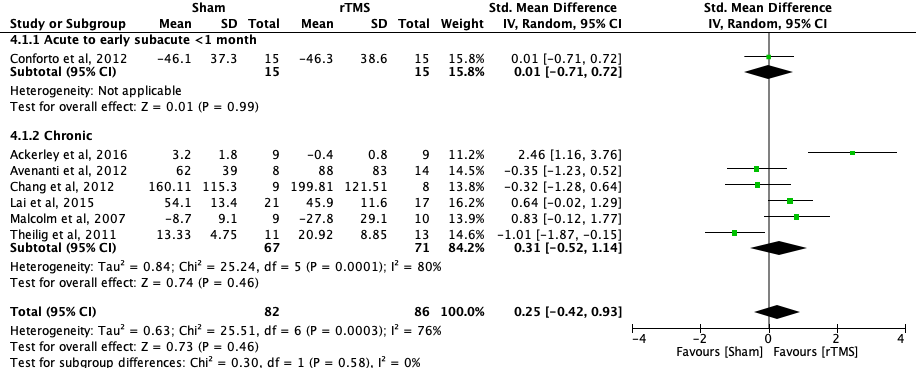

The standardized mean difference (SMD) and 95% confidence intervals (CIs); No studies within 1-3 and 3-6 months post-stroke subgroups.
**Supplementary Figure 4.** Effects of rTMS on the ICF Activity domain with 2-10 treatment sessions, comparing different treatment onset times. Estimates of effect size are shown with 95% CIs.

ICF Function (11-20 sessions)

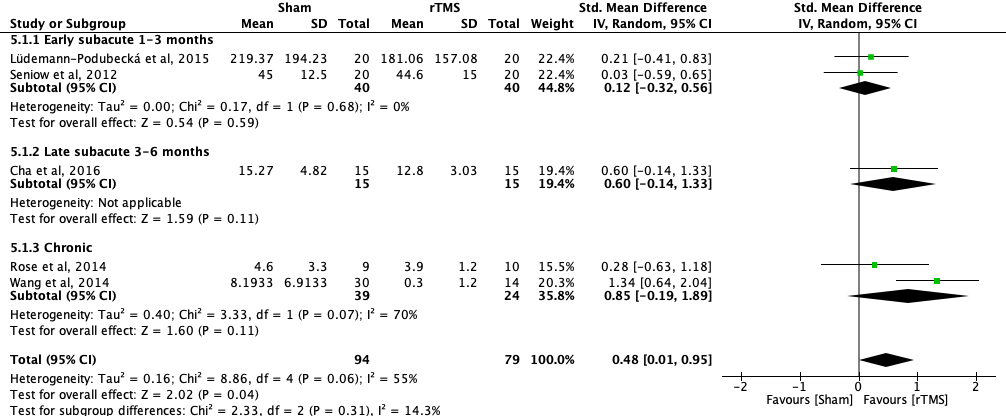

The standardized mean difference (SMD) and 95% confidence intervals (CIs); No studies within <1 month post-stroke subgroup.
**Supplementary Figure 5.** Effects of rTMS on the ICF Function domain with 11-20 treatment sessions, comparing different treatment onset times. Estimates of effect size are shown with 95% CIs.

**B** Additional therapy

ICF Function (rTMS + additional therapy)


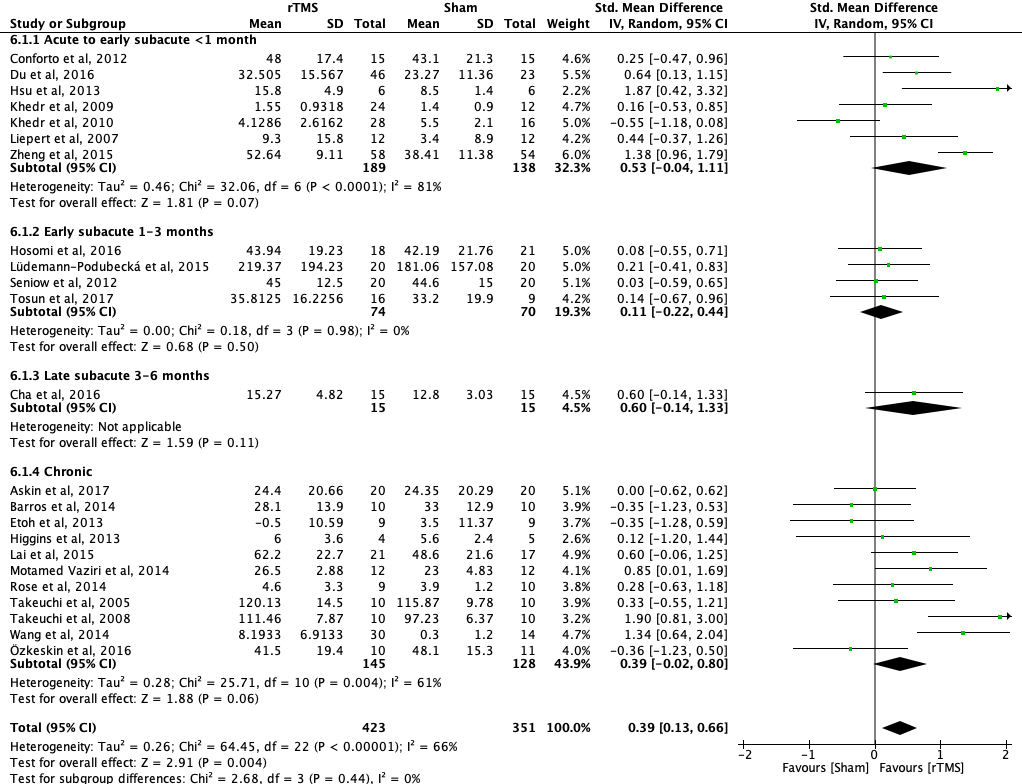

The standardized mean difference (SMD) and 95% confidence intervals (CIs)
**Supplementary Figure 6.** Effects of rTMS on the ICF Function domain with additional therapy, comparing different treatment onset times. Estimates of effect size are shown with 95% CIs.

ICF Function (rTMS + no additional therapy)


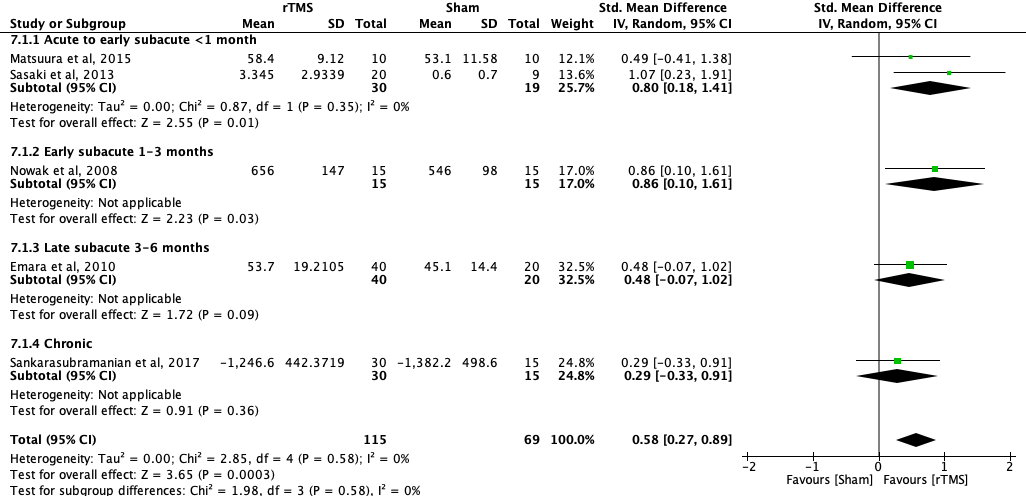

The standardized mean difference (SMD) and 95% confidence intervals (CIs)
**Supplementary Figure 7.** Effects of rTMS on the ICF Function domain without additional therapy, comparing different treatment onset times. Estimates of effect size are shown with 95% CIs.

ICF Activity (rTMS + additional therapy)


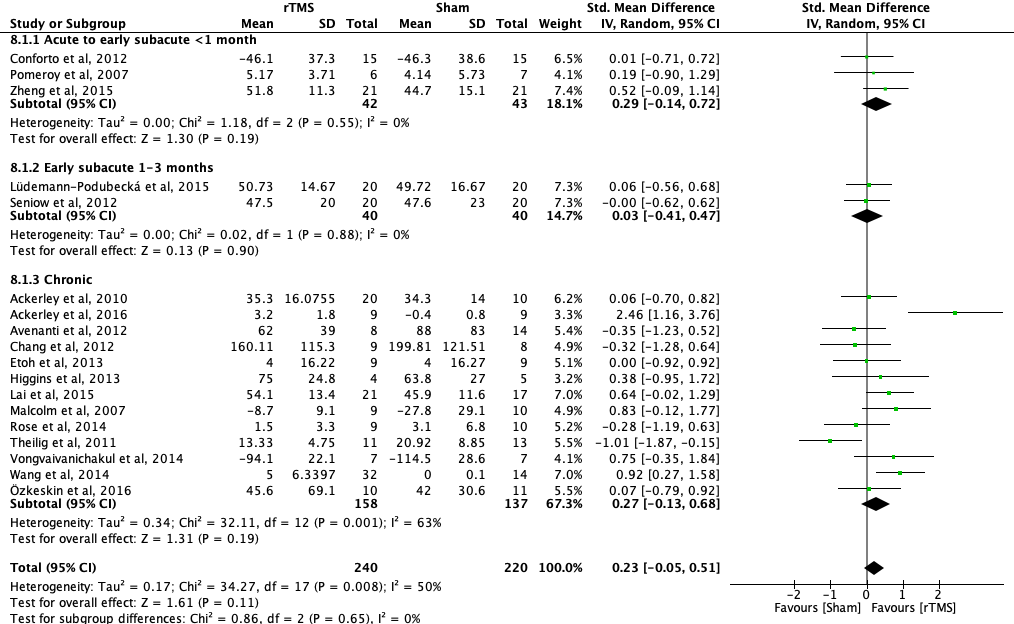

The standardized mean difference (SMD) and 95% confidence intervals (CIs); No studies within 3-6 months post-stroke subgroup.
**Supplementary Figure 8.** Effects of rTMS on the ICF Activity domain with additional therapy, comparing different treatment onset times. Estimates of effect size are shown with 95% CIs.

ICF Activity (rTMS + no additional therapy)

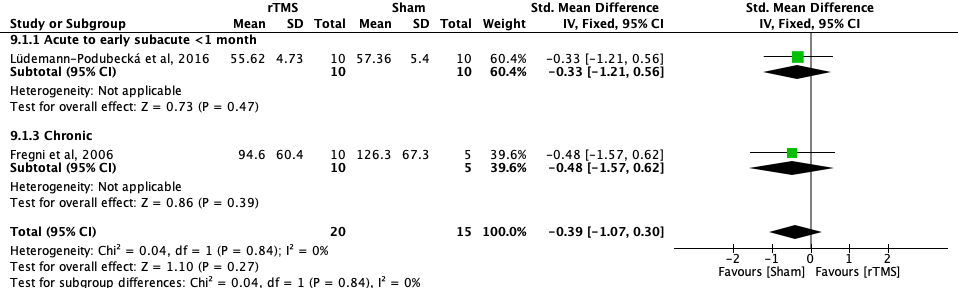

The standardized mean difference (SMD) and 95% confidence intervals (CIs); No studies within 1-3 and 3-6 months post-stroke subgroups.
**Supplementary Figure 9.** Effects of rTMS on the ICF Activity domain without additional therapy, comparing different treatment onset times. Estimates of effect size are shown with 95% CIs.

**C** rTMS frequency/site of stimulation

ICF Function (high frequency to the affected hemisphere)
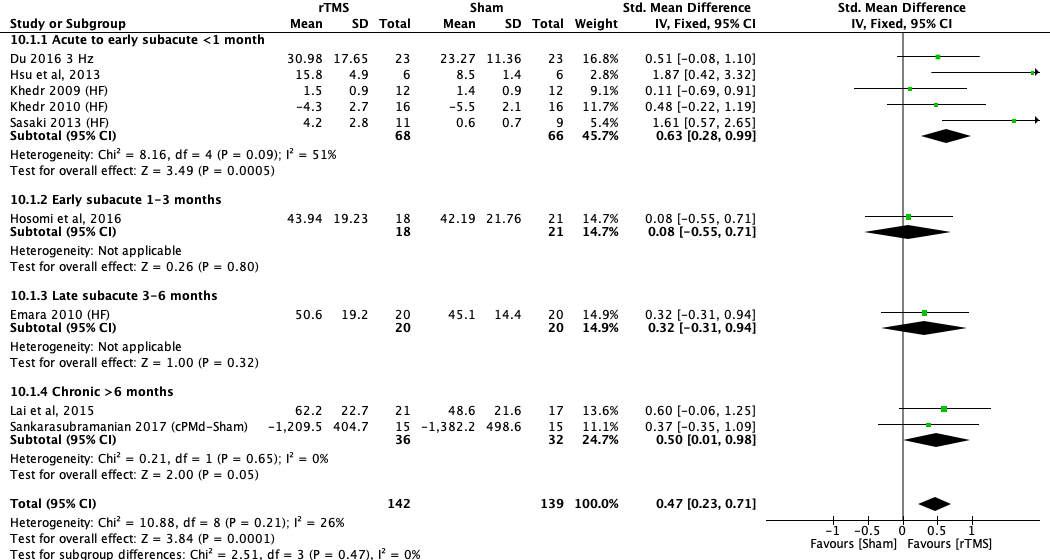

The standardized mean difference (SMD) and 95% confidence intervals (CIs)
**Supplementary Figure 10.** Effects of high frequency rTMS on the ICF Function domain, comparing different treatment onset times. Estimates of effect size are shown with 95% CIs.

ICF Function (low frequency to the unaffected hemisphere)

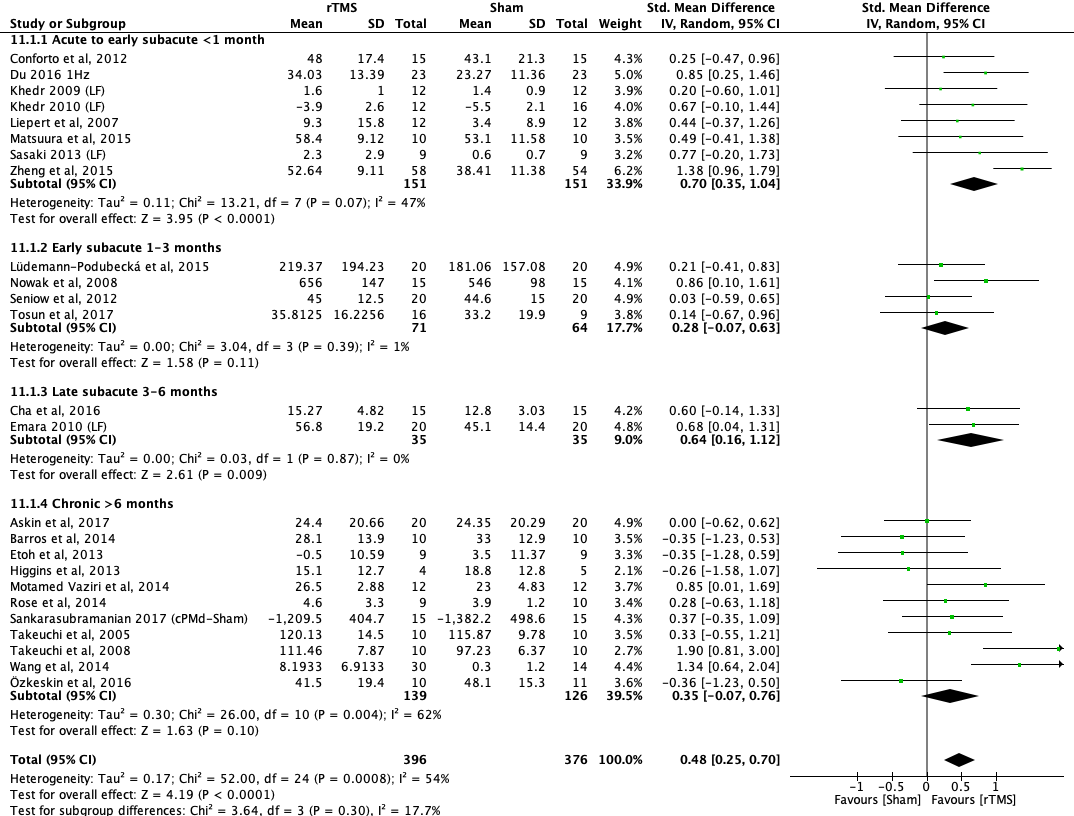

The standardized mean difference (SMD) and 95% confidence intervals (CIs)
**Supplementary Figure 11.** Effects of low frequency rTMS on the ICF Function domain, comparing different treatment onset times. Estimates of effect size are shown with 95% CIs.
